# A comprehensive review of *Schisandra chinensis* lignans: pharmacokinetics, pharmacological mechanisms, and future prospects in disease prevention and treatment

**DOI:** 10.1186/s13020-025-01096-z

**Published:** 2025-04-09

**Authors:** Danushiya Ehambarampillai, Murphy Lam Yim Wan

**Affiliations:** 1 Department of Laboratory Medicine, Division of Microbiology, Immunology and Glycobiology, Lund University, Lund, 221 84 Sweden; 2 School of Medicine, Pharmacy and Biomedical Sciences, Faculty of Science and Health, University of Portsmouth, PO1 2DT Portsmouth, United Kingdom

**Keywords:** *Schisandra chinensis*, Lignans, Pharmacological properties, Pharmacokinetics, Mechanism of action, Novel therapeutics

## Abstract

Lignans derived from *Schisandra chinensis* have attracted significant attention for their diverse pharmacological activities and clinical potential. This review presents a comprehensive analysis of the pharmacological properties of *Schisandra chinensis* lignans, including their antioxidant, anti-inflammatory, neuroprotective, hepatoprotective, antibacterial/viral, antidiabetic and anticancer effects. Their multifaceted mechanisms of action hold promise for therapeutic areas such as cancer, neurodegenerative diseases and metabolic disorders, aligning with urgent clinical needs. Additionally, this review explores the pharmacokinetics of these bioactive compounds, highlighting challenges in their absorption, distribution, metabolism and excretion, which impact their bioavailability. Recent advancements in drug delivery systems are discussed, highlighting their potential to enhance therapeutic efficacy in clinical settings. Furthermore, the synergistic effects of combining these lignans with other therapeutic agents are considered a strategy to increase their efficacy. Future research is imperative to identify additional active components and elucidate novel mechanisms of action, paving the way for expanded therapeutic applications and unlocking the full clinical potential of *Schisandra chinensis* in disease prevention and treatment.

## Introduction

*Schisandra chinensis* Turcz. (Baill.) is a medicinal plant that belongs to the family *Schisandra*ceae and is distributed in northeastern China, Japan, Korea and the Far East of Russia [1]. In traditional medicine, the fruits of *Schisandra chinensis* have been used to treat a wide range of disorders, including kidney infections, mental ailments, asthma, diabetes, coughs, spermatorrhoea, spontaneous sweating, thirst and insomnia [2, 3]. The World Health Organization added this plant to the International Pharmacopeia as a valuable medicinal plant in 2007 [4]. As the global demand for this plant has increased in the medical field, local farmers have expanded its cultivation [5].

**Fig. 1:**
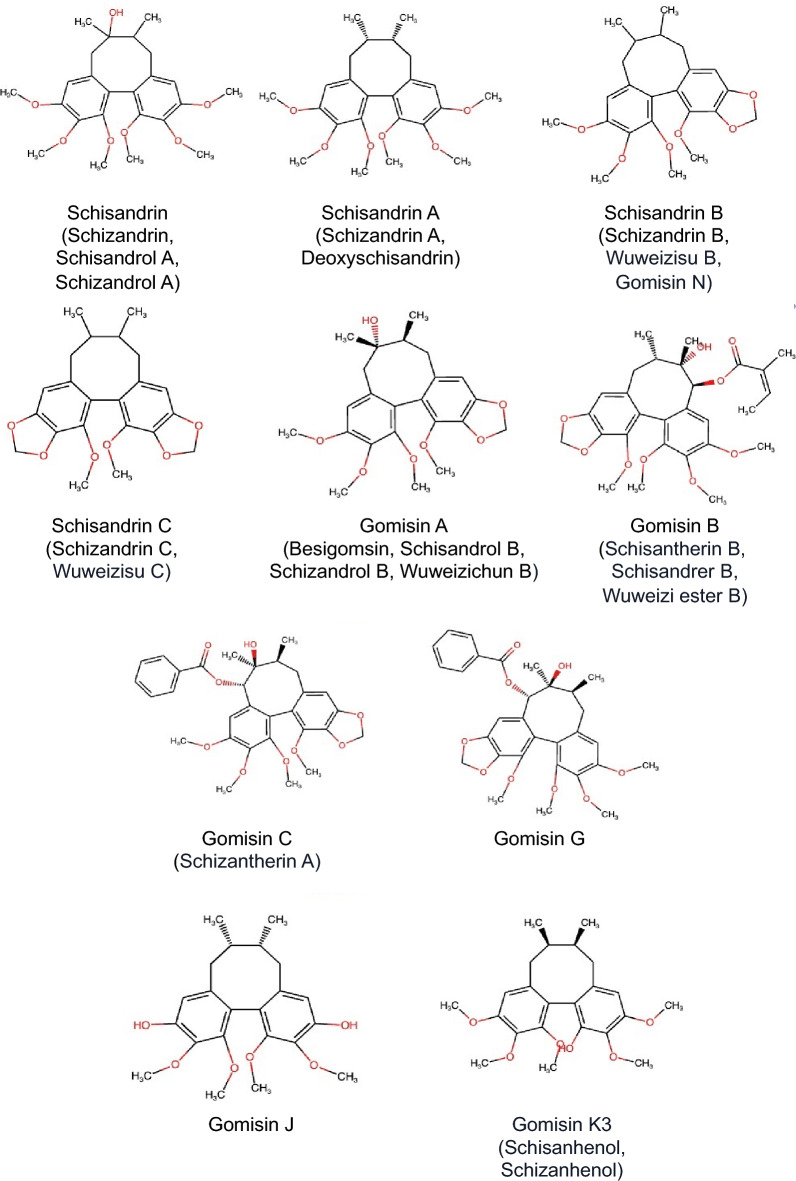
Chemical structures of major *Schisandra chinensis* lignans

**Fig. 2:**
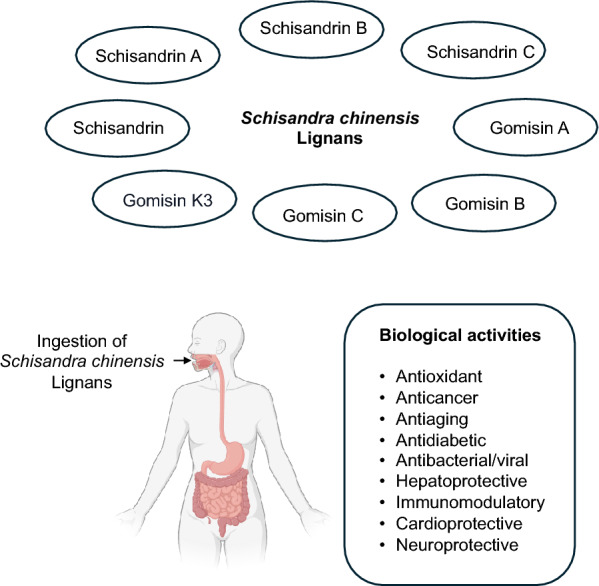
Major *Schisandra chinensis* lignans and their biological activities. The top panel illustrates key lignans found in *Schisandra chinensis*, including Schisandrin, Schisandrin A, Schisandrin B, Schisandrin C, Gomisin A, Gomisin B, Gomisin C and Gomisin K3. The bottom panel depicts the ingestion of *Schisandra chinensis* lignans and the major biological activities associated with these lignans, including antioxidant, anticancer, antiaging, antidiabetic, antibacterial/antiviral, hepatoprotective, immunomodulatory, cardioprotective and neuroprotective properties

The major bioactive compounds of *Schisandra chinensis* include phenolic acids, triterpenoids and lignans, along with other constituents, such as polysaccharides, essential oils and vitamins, found in various parts of the plant, including its flowers, leaves, seeds, stems and fruits [6]. Among these compounds, lignans, a unique class of polyphenolic compounds, have garnered particular interest because of their diverse biological activities, including the modulation of immune responses and the regulation of cellular signaling pathways. The most active lignans are dibenzocyclooctadiene derivative lignans such as schisandrin, schisandrin A, schisandrin B, schisandrin C, gomisin A, gomisin B, gomisin C, gomisin G, gomisin J and gomisin K3 (Fig. 1). These lignans have been extensively documented for their wide range of biological activities, including antioxidant, anticancer, antiaging, antidiabetic, antibacterial/viral, hepatoprotective, immunomodulatory, cardioprotective and neuroprotective effects (Fig. 2) [1, 7–11]. These findings have sparked considerable interest in exploring the therapeutic potential of *Schisandra chinensis*. Recent studies have particularly emphasized the significant anticancer potential of lignans. However, the clinical application of these compounds is often hindered by challenges such as poor solubility and limited bioavailability, necessitating the development of novel drug formulations to enhance their biological activity and clinical efficacy in treating various human diseases.

This review aims to provide a comprehensive analysis of the pharmacokinetics, pharmacological properties, mechanisms of action and future therapeutic prospects of *Schisandra chinensis* lignans. Particular attention is given to their potential as adjuvant therapies and the advancement of innovative drug delivery systems to increase their efficacy and expand their clinical applications.

## Methodology

### Study design

This review covers relevant and current literature on the pharmacokinetics, bioavailability, and bioactivity of *Schisandra chinensis* and its lignans. It also discusses the challenges and limitations associated with their use and explores strategies to enhance their therapeutic potential.

### Data collection

Data were collected by searching English articles in PubMed and Google Scholar. Moreover, data were collected from related past and recent research articles. The following keywords were used for the search of relevant articles: “*Schisandra chinensis*”, “schisandrin”, “schisandrin A”, “schisandrin B”, “schisandrin C”, “gomisin A”, “gomisin B”, “gomisin C”, “gomisin G”, “gomisin J” and “gomisin K3”, “pharmacokinetics”, “bioavailability”, “biological activity”, “antioxidant”, “anti-inflammatory”, “anticancer”, “antimicrobial”, “antiviral”, “antidiabetic”, “hepatoprotective”, “neuroprotective”, “drug delivery systems”, “combination”, “safety/toxicity” and “extraction methods”.

All related articles and abstracts published since 2000 were included. The selected articles were chosen based on their relevance to the objective of this review. Articles providing clear information on *Schisandra chinensis* and its lignans, their pharmacological effects, and their bioactivity and mechanisms of action were included. In total, 168 articles were included in this review.

To assess the drug-likeness and therapeutic potential of *Schisandra chinensis* lignans, SwissADME analysis (http://www.swissadme.ch/) was conducted for key compounds, including schisandrin, schisandrin A, schisandrin B, schisandrin C, gomisin A, gomisin B, gomisin C, gomisin G, gomisin J and gomisin K3.

## Pharmacokinetics and bioavailability of *Schisandra chinensis* lignans

Lignans are among the most prominent bioactive constituents of *Schisandra chinensis* and belong to a class of secondary plant metabolites formed through the oxidative dimerization of two phenylpropanoid units. To date, 86 lignans have been isolated and identified from *Schisandra chinensis*, with dibenzocyclooctadiene lignans representing the major bioactive constituents. These lignans exhibit significant structural diversity, contributing to their wide range of pharmacological activities (Fig. 2) [10].

The dibenzocyclooctadiene lignans of *Schisandra chinensis* are defined by their unique aryl-aryl bond and eight-membered carbocyclic ring, with variations in substitution patterns such as hydroxyl, methoxy, methylenedioxy and ester groups [10]. The pharmacokinetics and bioavailability of *Schisandra chinensis* lignans are critical factors influencing their therapeutic potential. However, lignans such as schisandrin A, schisandrin, gomisin A, gomisin C and schisanhenol often exhibit poor water solubility and undergo extensive first-pass metabolism, resulting in low systemic bioavailability.

After oral administration, these lignans are readily absorbed in the duodenum and jejunum, with subsequent absorption occurring in the colon and rectum [12]. Once absorbed, they are widely distributed throughout the body, particularly in the lungs and liver, followed by the heart, kidneys and spleen. Tissue distribution studies have shown that schisandrin B [13] and schisandrol B (gomisin A) are distributed throughout several tested tissues and accumulate mainly in the liver and kidneys [13, 14]. Interestingly, schisandrin and schisandrin A are distributed in the rat brain, suggesting that these *Schisandra chinensis* lignans can cross the blood‒brain barrier (BBB), supporting their neuroprotective potential [12]. By reaching neuronal tissues, these compounds may modulate oxidative stress, enhance antioxidant enzyme activity, and regulate neuroinflammatory pathways, making them promising candidates for mitigating neurodegenerative conditions and cognitive decline. Additionally, the absolute oral bioavailability of schisandrin B varies by sex, being approximately 55.0% in female rats and 19.3% in male rats [15]. A linear pharmacokinetic profile was observed within the tested oral range and showed extensive distribution in the ovaries and adipose tissue [16].

The phase I metabolic pathways of *Schisandra chinensis* lignans primarily involve demethylation and hydroxylation [12]. The liver plays a central role in lignan metabolism, with cytochrome P450 enzymes (CYP3A4 and CYP2C9) catalyzing their transformation into various metabolites [17, 18]. The induction of CYP by *Schisandra* extract may result from the activation of the pregnane X receptor (PXR), with schisandrin A, schisandrin B and gomisin B identified as the primary agonists responsible [17]. Notably, a more recent study revealed that gomisin A activated the PXR, potentially enhancing bile acid metabolism and efflux while increasing hepatic expression of *Cyp3a* in mice [19].

Lignans and their metabolites are primarily excreted via bile, feces or urine but are usually excreted in very low amounts [20]. In a study by Feng et al. the metabolic profiles of schisantherin A were investigated in vitro (rat liver microsomes) and in vivo (rat plasma, urine, and bile) via UPLC‒Q-TOF‒MS/MS. The analysis identified 56 metabolites in urine, 8 in bile, 19 in plasma and 5 in liver microsomes, highlighting both hepatic and extrahepatic biotransformation pathways [21]. Another study also showed that schisandrin B was primarily excreted as a metabolite, with minimal amounts in urine, bile and feces [16].

To assess the drug-likeness and therapeutic potential of *S. chinensis* lignans, SwissADME analysis was conducted for key compounds, including schisandrin, schisandrin A, schisandrin B, schisandrin C, gomisin A, gomisin B, gomisin C, gomisin G, gomisin J and gomisin K3 (Table 1). The results revealed that these lignans display notable structural diversity, reflected in their molecular weights, which range from 384.42 g/mol (schisandrin C) to 536.57 g/mol (gomisin C and gomisin G). The number of heavy atoms also varies, with smaller lignans, such as schisandrin C, containing 28 heavy atoms, and larger lignans, such as gomisin C and gomisin G, containing 39 heavy atoms, reflecting differences in molecular size.

**Table 1:** ADME analysis of common *Schisandra* lignans via Swiss ADME (http://www.swissadme.ch/)

	Schisandrin	Schisandrin A	Schisandrin B	Schisandrin C	Gomisin A	Gomisin B	Gomisin C	Gomisin G	Gomisin J	Gomisin K3
Formula	C_24_H_32_O_7_	C_24_H_32_O_6_	C_23_H_28_O_6_	C_22_H_24_O_6_	C_23_H_28_O_7_	C_28_H_34_O_9_	C_30_H_32_O_9_	C_30_H_32_O_9_	C_22_H_28_O_6_	C_23_H_30_O_6_
Molecular weight	432.51 g/mol	416.51 g/mol	400.46 g/mol	384.42 g/mol	416.46 g/mol	514.56 g/mol	536.57 g/mol	536.57 g/mol	388.45 g/mol	402.48 g/mol
#Heavy atoms	31	30	29	28	30	37	39	39	28	29
Fraction Csp3	0.50	0.50	0.48	0.45	0.48	0.46	0.37	0.37	0.45	0.48
#Rotatable bonds	6	6	4	2	4	7	7	7	4	5
#H-bond acceptors	7	6	6	6	7	9	9	9	6	6
#H-bond donors	1	0	0	0	1	1	1	1	2	1
Molar Refractivity	119.08	117.88	110.96	104.03	112.15	137.00	142.96	142.96	108.94	113.41
TPSA	75.61 Å^2^	55.38 Å^2^	55.38 Å^2^	55.38 Å^2^	75.61 Å^2^	101.91 Å^2^	101.91 Å^2^	101.91 Å^2^	77.38 Å^2^	66.38 Å^2^
iLOGP	3.91	4.25	4.09	3.80	3.81	3.88	4.10	4.31	3.60	3.91
XLOGP3	4.03	5.26	5.13	5.00	3.90	4.56	5.02	5.02	4.61	4.93
WLOGP	3.89	4.78	4.49	4.20	3.60	4.29	4.63	4.63	4.17	4.47
MLOGP	1.77	2.57	2.76	2.96	1.96	2.08	2.31	2.31	2.14	2.36
Silicos-IT Log P	4.67	5.16	4.88	4.62	4.39	4.91	5.07	5.07	4.06	4.61
Consenus Log P	3.65	4.40	4.27	4.11	3.53	3.94	4.23	4.27	3.72	4.06
ESOL Class	Moderately soluble	Moderately soluble	Moderately soluble	Moderately soluble	Moderately soluble	Moderately soluble	Poorly soluble	Poorly soluble	Moderately soluble	Moderately soluble
Ali Class	Moderately soluble	Poorly soluble	Poorly soluble	Moderately soluble	Moderately soluble	Poorly soluble	Poorly soluble	Poorly soluble	Moderately soluble	Poorly soluble
Silcos-IT class	Poorly soluble	Poorly soluble	Poorly soluble	Poorly soluble	Poorly soluble	Poorly soluble	Poorly soluble	Poorly soluble	Moderately soluble	Poorly soluble
GI absorption	High	High	High	High	High	High	High	High	High	High
BBB permeant	Yes	Yes	Yes	Yes	Yes	No	No	No	No	Yes
P-gp substrate	No	No	No	No	No	Yes	Yes	Yes	No	No
CYP1A2 inhibitor	No	No	No	Yes	No	No	No	No	No	No
CYP2C19 inhibitor	No	No	Yes	Yes	No	No	No	No	No	No
CYP2C9 inhibitor	No	No	Yes	Yes	Yes	No	Yes	Yes	No	No
CYP2D6 inhibitor	Yes	Yes	Yes	Yes	Yes	Yes	Yes	Yes	No	No
CYP3A4 inhibitor	No	No	No	No	Yes	No	Yes	Yes	No	No
Lipinski #violations	Yes; 0	Yes; 0	Yes; 0	Yes; 0	Yes; 0	Yes; 1 MW > 500	Yes; 1 MW > 500	Yes; 1 MW > 500	Yes; 0	Yes; 0
Bioavailability Score	0.55	0.55	0.55	0.55	0.55	0.55	0.55	0.55	0.55	0.55

Key determinants of molecular flexibility, such as the fraction of sp^3^ hybridized carbons (Csp^3^) and the number of rotatable bonds, vary significantly among lignans. For example, schisandrin C has a Csp^3^ fraction of 0.37 and two rotatable bonds, whereas gomisin C has a Csp^3^ fraction of 0.37 but seven rotatable bonds, indicating greater molecular flexibility. Additionally, the total polar surface area (TPSA), a crucial determinant of hydrogen bonding and solubility, varies significantly among lignans, ranging from 55.38 Å^2^ (schisandrin B and schisandrin C) to 101.91 Å^2^ (gomisin B, gomisin C, and gomisin G). Higher TPSA values in gomisin B and gomisin C suggest greater potential for interactions with hydrophilic biological targets but may reduce permeability across lipid membranes.

Lipophilicity, a critical property for drug permeability, is moderate to high across all lignans, as evidenced by their consensus LogP values, ranging from 3.53 (gomisin A) to 4.40 (schisandrin A). While this lipophilicity enables lignans to cross cell membranes, it may also limit their water solubility. Most lignans are categorized as poorly soluble (e.g., gomisin B and gomisin C), which may affect their bioavailability unless advanced formulation strategies are employed. Schisandrin and schisandrin A, which have moderate solubilities, are exceptions to this trend.

**Fig. 3:**
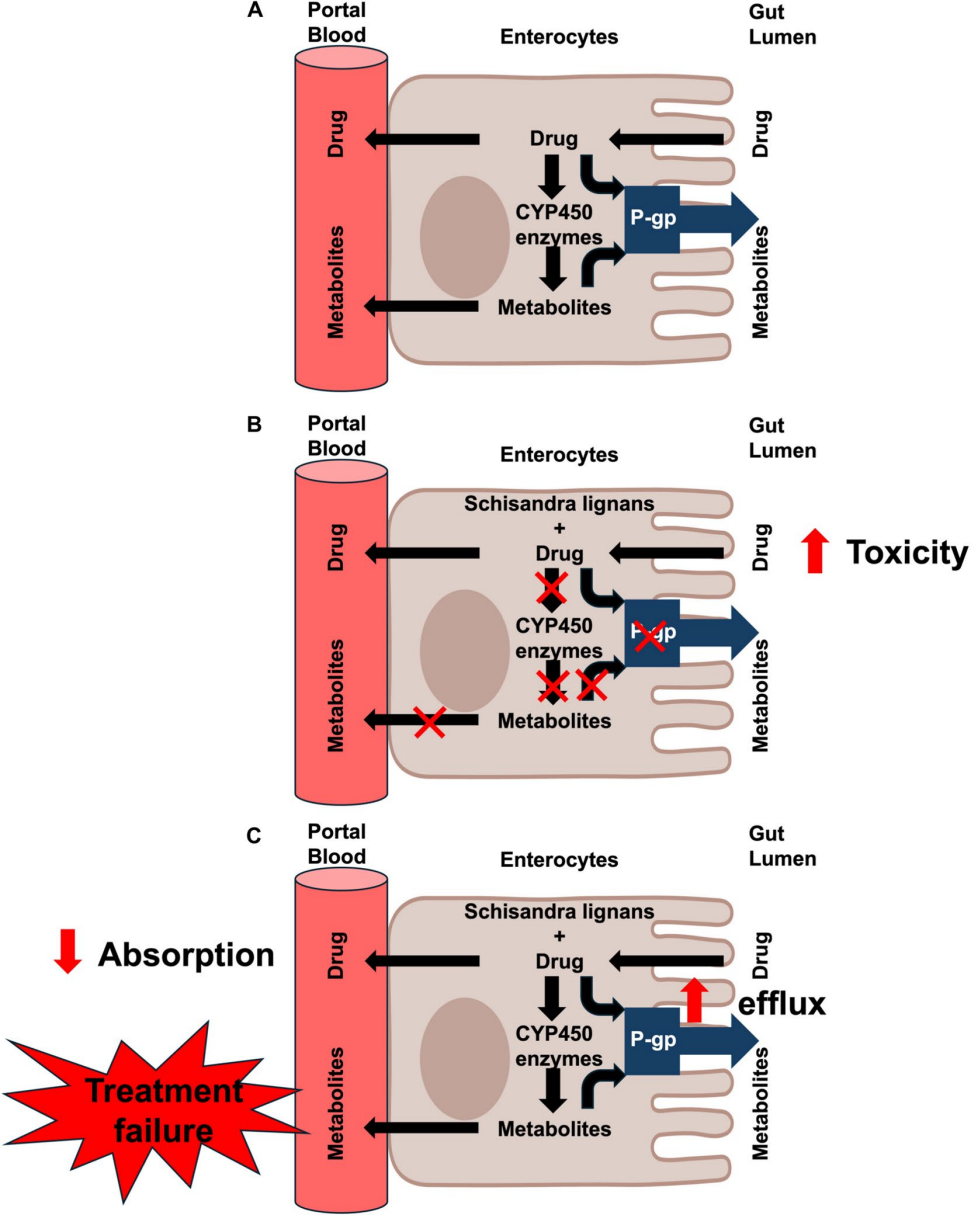
Potential effect of *Schisandra* lignans on drug metabolism in enterocytes. **A** Under normal conditions, drugs are absorbed into enterocytes, where they undergo metabolism by cytochrome P450 (CYP450) enzymes, resulting in metabolite formation. Some drugs are also subject to efflux by P-glycoprotein (P-gp), which pumps them back into the gut lumen, limiting their bioavailability. **B** When *Schisandra* lignans inhibit CYP450 enzymes and P-gp, drug metabolism is reduced, leading to higher drug concentrations in the bloodstream. This decreased metabolism and impaired efflux can result in increased drug bioavailability and toxicity. **C** Alternatively, *Schisandra* lignans may induce P-gp expression, leading to enhanced drug efflux back into the gut lumen. This reduces drug absorption and bioavailability, potentially resulting in treatment failure due to insufficient therapeutic drug levels in the bloodstream. Arrows indicate drug movement, metabolism, and efflux

From a pharmacokinetic perspective, all lignans demonstrate high gastrointestinal (GI) absorption, supporting their oral administration potential. Most compounds, including schisandrin A, schisandrin B and gomisin A, are capable of crossing the BBB, highlighting their potential for neurological applications. However, certain lignans, such as gomisin B, gomisin C and gomisin G, are not BBB permeable, which may limit their use as central nervous system targets. Additionally, some lignans are substrates or inhibitors of key cytochrome P450 enzymes, including CYP2D6 and CYP2C9, potentially competing with other drugs for metabolism. This competition can lead to elevated plasma and tissue drug concentrations, increasing the risk of toxicity (Fig. 3A, B).

Interestingly, lignans such as gomisin B, gomisin C and gomisin G are p-glycoprotein (P-gp) substrates, which may significantly impact their pharmacokinetics and therapeutic potential. As P-gp substrates, these compounds can undergo active efflux from intestinal cells back into the gut lumen, reducing their absorption and systemic bioavailability following oral administration [22]. In cancer therapy, P-gp plays a crucial role in multidrug resistance (MDR) by actively pumping out chemotherapeutic agents, thereby reducing their intracellular concentration and leading to treatment failure (Fig. 3C) [23]. If gomisin B, C and G are rapidly effluxed from cancer cells, their effectiveness as anticancer agents may be compromised. However, if these lignans modulate P-gp activity—either by inhibiting or inducing its function—they could influence the efficacy of chemotherapy. Additionally, if they compete with other P-gp substrates, they may impact the pharmacokinetics of co-administered drugs, potentially altering their therapeutic effects. In contrast, lignans such as schisandrin, schisandrin A and schisandrin B are not P-gp substrates, which enhances their potential for intracellular accumulation and targeted therapeutic effects, contributing to their increased efficacy.

The bioavailability score for all lignans is 0.55, which is consistent with moderate drug likeness. However, larger lignans such as gomisin B, gomisin C and gomisin G violate Lipinski's Rule of Five due to their molecular weights exceeding 500 g/mol, which may impact their drug development prospects.

Overall, the data highlight the pharmacokinetic challenges of *Schisandra chinensis* lignans, such as poor solubility and extensive metabolism, necessitating advanced formulation strategies to optimize their therapeutic potential. Despite these limitations, their structural diversity, high GI absorption, and ability to cross the BBB make them compelling candidates for further drug development. Future research should focus on enhancing bioavailability and exploring their multifaceted biological activities to unlock their full therapeutic potential.

## Biological activity of *Schisandra chinensis*

*Schisandra chinensis* is a remarkable medicinal plant known for its diverse array of bioactive compounds and rich chemical composition, which collectively offer significant health-promoting potential. Central to its biological efficacy are its bioactive lignans, which exhibit a wide spectrum of pharmacological activities, including antioxidant, anti-inflammatory, antimicrobial/viral, hepatoprotective, neuroprotective, antidiabetic and anticancer properties (Fig. 2 and Table 2). These properties underscore the versatility of plants and their potential for therapeutic applications across multiple disease areas.

**Table 2:** Summary of the pharmacological activities, effects and/or mechanisms of action of key *Schisandra* lignans

Pharmacological activity	Experimental model	Dosage	Effects and/or mechanisms of action	References
*Schisandrin*				
–	Healthy Sprague–Dawley (SD) rats	Wu Wei Zi (*Schisandra chinensis* Baill) and Gan Cao (*Glycyrrhiza uralensis* Fisch) containing Schisandrin, oral gavage for 6 days	• ↑ Pregnane X receptor (PXR), ↑ CYP3A, CYP2C, MRP2 • ↑ Metabolism of coadministered warfarin	[17]
Anticancer	Human promyelocytic leukemia MDR HL60/ADR and HL60/MRP cells	Up to 20 μg/ml, varied durations	• Reverse drug resistance to vincristine, daunorubicin, and VP-16 • ↓ P-gp and MRP1, ↑ apoptosis	[98]
	Human colon carcinoma Caco-2 cells	10–50 μM, up to 4 h	• Reversed P-gp-mediated MDR, ↓ drug efflux in cancer cells	[97]
Neuroprotective antioxidant	Amyloid-beta1-42 (Aβ1–42)-induced memory impairment in mice	4, 12, and 36 mg/kg/b.w., intragastric infusion for 14 days	• ↑ Superoxide dismutases (SOD) and glutathione peroxidase (GSH-px) activities, glutathione (GSH) level, and GSH/ oxidized glutathione (GSSG) ratio, ↓ malondialdehyde (MDA) and GSSG • ↑ Memory	[57]
Anti-inflammatory	Cerebral cortical cells from neonatal SD rats Healthy kunming mice	10, 20, 40, or 80 μmol/l Sch for 48 h 5, 15, or 45 mg/kg/d, i.g. for 14 days	• ↓ Neurotoxicity, ↑ cerebral cortical cell viability, ↑ cognitive performance and histological integrity	[56]
	6-Hydroxydopamine (6-OHDA)-induced Parkinson’s mice	10–30 mg/kg, via drinking water for 14 days	• ↓ Neuronal inflammation and oxidative stress, and ↑ neuron survival • ↑ PI3K/Akt pathway, ↓ IKK/IκBα/NF-κB pathway	[55]
Hepatoprotective	Acetaminophen (APAP)-induced liver injury in C57BL/6 mice	200 mg/kg/day, orally, seven doses at 12-h intervals before APAP treatment	• ↓ APAP-induced liver damage, and alanine aminotransferase (ALT)/ aspartate transaminase (AST) levels • ↑ Total and mitochondrial GSH • ↓ CYP2E1, CYP1A2, and CYP3A11 activity, NAPQI-GSH formation	[51]
	Lithocholic acid-induced cholestasis in C57BL/6 J mice	100 mg/kg/day, orally for 7 days	• Protected against cholestasis through PXR activation, ↑ hPXR-regulated genes CYP3A4, UGT1A1, and OATP2 • ↓ Liver necrosis, ALT, AST, and ALP activity • ↓ Serum total bile acids and bilirubin • ↑ Bile acid metabolism, efflux from liver into intestine/feces	[146]
Anti-diabetic	High fat diet (STZ/HFD) induced diabetic nephropathy (DN) in C57BL/6 J mice	10 and 20 mg/kg/day, i.g. for 8 weeks	• ↓ TGF-β1-mediated renal fibrosis • ↓ Inflammation via modulation of PI3K/Akt and NF-κB pathways	[76]
Anti-inflammatory				
*Schisandrin A*				
Neuroprotective	Glutamate-induced neurotoxicity in primary rat cortical cells	0.1–5 µM for 24 h	• ↓ Oxidative stress, nitric oxide (NO) overproduction, ↑ GSH-px, GSH	[27]
–	Healthy SD rats	Wu Wei Zi (*Schisandra chinensis* Baill) and Gan Cao (*Glycyrrhiza uralensis* Fisch) containing Schisandrin A, oral gavage for 6 days	• ↑ PXR, ↑ CYP3A, CYP2C, MRP2 • ↑ Metabolism of coadministered warfarin	[17]
Anticancer	Human colon adenocarcinoma LoVo cells	25–250 μM for 24, 48, and 72 h	• ↑ Apoptosis	[82]
	Human colon adenocarcinoma DLD1, RKO, SW480, SW620 cells	150, 125, 100, 75, and 50 μM for 72 h	• Bind directly with HSF1—> ↓ HSF1, ↑ apoptosis and cell cycle arrest	[86]
Anti-inflammatory	IL-1β stimulated human osteoarthritis chondrocytes	0–47 μg/ml for 24 h	• ↓ NO, PGE2, and TNF-α production • ↓ MMP1, MMP3, and MMP13 expression • ↓ NF-κB and MAPKs pathways	[34]
Antiviral	Human hepatoma Huh-7 and baby hamster kidney fibroblasts BHK-21 Dengue virus-infected ICR suckling mice	0–50 μM for 3 days 5 or 10 mg/kg, every 2 days at 1, 3, and 5 days post infection	• ↓ Symptoms and mortality in infected mice • ↓ Viral replication, ↑ antiviral interferon responses via STAT1/2 pathway	[44]
Anti-inflammatory	LPS-treated murine macrophages RAW 264.7 cells	0–200 μM for 24 h	• ↓ NO & PGE-2, ↓ iNOS and COX-2 at mRNA and protein levels • ↓ TNF-α, IL-1β • ↓ NF-κB, MAPK, and PI3K/Akt pathways, ↑ Nrf2/HO-1 pathway	[33]
Antioxidant				
Hepatoprotective	Acetaminophen (APAP)-induced liver injury in C57BL/6 mice	200 mg/kg/day, orally, seven doses at 12-h intervals before APAP treatment	• ↓ APAP-induced liver damage, and ALT/AST levels • ↑ Total and mitochondrial GSH • ↓ CYP2E1, CYP1A2, and CYP3A11 activity, NAPQI-GSH formation	[51]
	Lithocholic acid-induced cholestasis C57BL/6 J mice	100 mg/kg/day, orally for 7 days	• Protected against cholestasis through PXR activation, ↑ hPXR-regulated genes CYP3A4, UGT1A1, and OATP2 • ↓ Liver necrosis, ALT, AST, and ALP activity • ↓ Serum total bile acids and bilirubin • ↑ Bile acid metabolism, efflux from liver into intestine/feces	[146]
Anti-inflammatory	LPS-induced mastitis in C57BL/6 mice and mouse mammary epithelial cells	32 mg/kg, i.p., 12 h + 12 h Up to 100 μM for 1 h	• ↑ Nrf2 pathway, ↑ autophagy • ↓ mTOR, ↑ AMPK-ULK1 pathway	[35]
Anti-inflammatory	Diabetic nephropathy in C57BL/6 mice d-glucose treated human renal glomerular endothelial cells	25, 50 or 100 mg/kg for 8 weeks 25, 50, 100 μM for 2 h	• ↓ High glucose-induced ferroptosis, ROS-mediated pyroptosis by mitochondrial damage • ↓ AdipoR1 ubiquitination • ↑ AdipoR1/AMPK, ↓ TXNIP/NLRP3 pathways	[75]
Antioxidant				
*Schisandrin B*				
Hepatoprotective	Carbon tetrachloride (CCl4)-intoxicated BALB/c mice	1 mmol/kg/day, i.g. for 3 days	• ↓ CCl4-induced increase in plasma ALT activity, plasma SDH activity • ↑ Mitochondrial GSH redox status, mitochondrial glutathione reductase (GR) activity	[50]
	Menadione-induced hepatotoxicity in SD rats	1 mmol/kg/day, i.g. for 3 days	• ↓ Plasma ALT activity, hepatic MDA, ↑ DT-diaphorase (DTD) activity	[147]
	Healthy BALB/c mice	0.5 to 2 mmol/kg/day, i.g. for 3 days	• Protected against TNFα-induced apoptosis • ↑ Hepatic Hsp70 level	[148]
	Acetaminophen (APAP)-induced liver injury in C57BL/6 mice	200 mg/kg/day, orally, seven doses at 12-h intervals before APAP treatment	• ↓ APAP-induced liver damage, and ALT/AST levels • ↑ Total and mitochondrial GSH • ↓ CYP2E1, CYP1A2, and CYP3A11 activity, NAPQI-GSH formation	[51]
	Lithocholic acid-induced cholestasis in C57BL/6 J mice	100 mg/kg/day, orally for 7 days	• Protected against cholestasis through PXR activation, ↑ hPXR-regulated genes CYP3A4, UGT1A1, and OATP2 • ↓ Liver necrosis, ALT, AST, and ALP activity • ↓ Serum total bile acids and bilirubin • ↑ Bile acid metabolism, efflux from liver into intestine/feces	[146]
	Human umbilical cord mesenchymal stem cells (UC-MSCs)-derived hepatocyte-like cells (HLCs) CCl4-intoxicated C57BL/6 J mice	0, 1, 10, 25, 50, 100, and 200 μM for 72 h HLC transplantation, sacrifice after 7 and 14 days	• ↑ Hepatic differentiation and maturation of UC-MSCs • ↑ JAK2/STAT3 pathway • ↓ Liver fibrosis	[49]
Neuroprotective	Glutamate-induced neurotoxicity in primary rat cortical cells	0.1–5 µM for 24 h	• ↓ Intracellular calcium overload, oxidative stress, NO overproduction, ↑ GSH-px, GSH	[27]
	Scopolamine-induced memory deficits in BALB/c mice	10, 25 or 50 mg/kg/day, orally for 7 days	• Improved memory impairment, behavioral tasks • ↑ Acetylcholine (ACh), ↓ Acetylcholinesterase (AChE) • ↓ MDA, ↑ GSH, SOD, and GPx	[29]
	Focal cerebral ischemia in SD rats	10 or 30 mg/kg, i.p, 30 min before onset of ischemia and 2 h after surgery	• ↓ Infarct volumes • ↓ TNF-α and IL-1β, and degradation of metalloproteinase (MMP)-2 and MMP-9	[65]
	LPS-induced rat primary microglia-enriched cultures	2.5, 5, 10 or 20 μM for 24 h or 10 min prior to LPS treatment	• ↓ NO, TNF-α, PGE2, IL-1β, IL-6 • ↓ TLR4-dependent MyD88/IKK/NF-κB pathway • ↑ ROS, NADPH oxidase activity	[37]
	Intracerebroventricular (ICV)-infused Aβ-induced neuronal dysfunction in SD rats	25 and 50 mg/kg, orally via tubing to the stomach for 26 days	• ↓ Aβ memory impairment, ↓ neuronal death • ↑ ACh, ↓ AChE • ↓ Oxidative and nitrosative stresses, inflammatory markers e.g. inducible nitric oxide syntheses (iNOS), COX-2, IL-1β, IL-6, and TNF-α, and DNA damage • ↓ Receptor for advanced glycation end products (RAGE), NF-κB, MAKPs • ↑ HSP70 and beclin	[61]
	LPS-stimulated BV2 microglia	0–50 μM for 24 h	• ↓ TNF-α, IL-6, IL-1β, and PGE2 • ↓ NF-κB, ↑PPAR-γ	[64]
Anticancer	Human erythroleukemia K562/Adr, breast cancer MCF-7/Adr, epidermoid carcinoma KBv200, and Bcap37/Ad	10 μg/ml for 3 days	• ↓ MDR, P-gp	[92]
	Human promyelocytic leukemia HL60/ADR, HL60/MRP cells	0–100 μM, varied durations	• ↓ MDR, MRP1, and P-gp	[91]
	Human hepatoblastoma HepG2 cells	40, 80, 160, 320 μM for 24 h	• ↑ Apoptosis, ↑ BAX and BCL-2	[81]
	Human promyelocytic leukemia U937 cells	0–150 μM for 48 h	• ↓ Cell proliferation, ↑ apoptosis • ↓ BCL-2, mitochondrial membrane potential (MMP), a release of cytochrome c from mitochondria to cytosol • ↑ Proteolytic activation of caspase-9 and -3, and degradation of poly(ADP-ribose) polymerase	[149]
	Human colon cancer HCT116 cells	5–20 μM for 24–72 h	• ↑ Cell cycle arrest, apoptosis • ↓ Wnt/β-catenin signaling by preventing β-catenin from binding to DNA at TCF binding sites • ↓ Cyclin D1, Cdk2, and Cdk4 • ↑ Cleavage of PARP and caspase-3	[150]
	Colon adenocarcinoma (LoVo)	25 to 250 μM for 24, 48 and 72 h	• ↑ apoptosis, cell cycle arrest correlated with tubulin polymerization	[82]
	Human breast cancer MCF-7, A2780 human ovarian cancer and DOX-resistant A2780/DOX cells	5, 10, and 20 μM for 48 h	• ↓ DOX resistance • ↓ P-gp expression and activity • ↓ Survivin, ↑ chymotryptic activity of proteosome	[93]
	Human Triple-negative breast cancer (TNBC) MDA-MB-231, BT-549, and MDA-MB-468 Athymic BALB/c nu/nu mice xenograft with MDA-MB-231	Up to 100 μM for 12 or 18 h 50 or 100 mg/kg, i.p., twice daily for 24 days	• ↑ Apoptosis, cell cycle arrest, ↓ cell viability, cell migration • ↓ STAT3	[89]
	TNBC cell lines and patient-derived TNBC cells	5 to 20 μM for 24 or 48 h	• ↓ Cell growth, migration and invasion • ↓ Inflammasome activation, IL-1β	[95]
	Human colon carcinoma HCT116, HT29, SW480, SW620, Caco-2, LS174T cells BALB/c nude mouse xenograft with HCT116 cells	Up to 100 μM for 24 or 48 h 50 mg/kg, oral gavage, once every other day for 14 days	• ↑ Apoptosis, cell cycle arrest, ↓ cell proliferation • ↓ CHOP	[84]
Cardioprotective	Chronic cardiotoxicity in SD rats Breast cancer 4T1, and sarcoma S180	50 µM for 72 h 50 mg/kg, i.g., 2 h prior to DOX treatment weekly over 5 weeks	• ↓ DOX-induced loss of cardiac function, damage of cardiomyocytic structure • ↑ DOX cytotoxicity in cancer cells • ↓ Lung metastasis in Sch B + DOX treated rats	[94]
Anticancer				
Anti-inflammatory	LPS-stimulated murine macrophage RAW 264.7 cells	1–10 µM for 24 h	• ↓ TNF-α, IL-6, and IL-1β mRNA and protein • ↓ NO secretion • ↓ p38 MAPK, ERK1/2, JNK phosphorylation	[151]
	LPS-induced human umbilical vein endothelial cells	Up to 40 μM for 24 h	• ↓ TNF-α, IL-8, VCAM-1, and ICAM-1 • ↓ NF-κB, ↑ Nrf2 and HO-1	[39]
	Allergic asthma in BALB/c mice	15, 30 or 60 mg/kg, oral gavage, 1 h before ovalbumin (OVA) challenge, once a day for 2 months	• ↓ IgE, pathological injury • ↑ Nrf2, ↓ NF-kB caused by OVA	[41]
Anti-inflammatory	UVB-irradiated human dermal fibroblasts and melanoma cells	0.1, 1 and 10 μM for up to 24 h	• ↑ SOD, ↓ MDA and ROS • ↓ COX-2, IL-6, IL-18, MMP-1, and degradation of collagens	[31]
Antioxidant				
Anti-diabetic	High fat diet (HFD)-induced obese C57BL/6 mice Mouse C2C12 myoblasts	20 mg/kg/day, orally for 6 weeks 10, 50, or 100 μM for 6 or 12 h	• ↑ AMPK/Akt pathway • ↑ Glucose uptake, mitochondria biogenesis and fatty acid oxidation • ↓ Hyperglycemia, ↑ glucose tolerance	[74]
	Human kidney 293 (HEK293), mouse insulin islet (β-TC-6) cells HFD combined with streptozotocin (STZ) injection-induced T2DM in C57BL/6 mice	20 μmol/l, various durations 50,100, or 200 mg/kg/day, oral gavage for 6 weeks	• ↑ Insulin secretion via GLP-1R/cAMP/PKA pathway	[73]
*Schisandrin C*				
Neuroprotective	Glutamate-induced neurotoxicity in primary rat cortical cells	0.1–5 µM for 24 h	• ↓ Intracellular calcium overload, oxidative stress, NO overproduction, ↑ GSH-px, GSH	[27]
	Bacterial lipoteichoic acid (LTA)-stimulated mouse primary microglia, mouse BV2 microglial cells	0–20 µM, varied duration	• ↑ HO-1, NqO-1 via ↑ cAMP/PKA/CREB, Nrf-2 pathways • ↓ PGE2, NO, ROS, iNOS, COX-2, MMP, NF-κB, AP-1, JAK/STATs, and MAPKs	[67]
	Aβ1–42-induced Alzheimer’s Kunming mice	15 or 150 µg/kg/day, by alloy-steel tube for 5 days	• Improved short-term or working memory • ↑ SOD, GSH-px, GSH in hippocampus and cerebral cortex • ↓ Total ChE	[66]
	Serum and glucose deprivation (SGD) injury in SH-SY5Y cells	2.5–5 µg/ml for 23.5 h	• ↓ NF-κB, IL-1β, and NLRP3 inflammasome activation • ↓ Apoptosis, caspase-1 and caspase-3, JNK/MAPK pathway	[69]
Anticancer	Human hepatocellular carcinoma Bel-7402, human breast cancer Bcap37, human nasopharyngeal carcinoma KB-3–1, human endothelial ECV-304, and human liver QSG-7701 cells	Up to 200 μM for 24 or 48 h	• ↑ Apoptosis	[152]
Anti-inflammatory	LPS-stimulated murine macrophage RAW 264.7 cells	1–10 µM for 24 h	• ↓ TNF-α, IL-6, and IL-1β mRNA and protein • ↓ NO secretion • ↓ p38 MAPK, ERK1/2, JNK phosphorylation	[151]
Hepatoprotective	Acetaminophen (APAP)-induced liver injury in C57BL/6 mice	200 mg/kg/day, orally, seven doses at 12-h intervals before APAP treatment	• ↓ APAP-induced liver damage as seen in histological analysis and ALT/AST levels • ↑ Total and mitochondrial GSH • ↓ CYP2E1, CYP1A2, and CYP3A11 activity, NAPQI-GSH formation	[51]
	Lithocholic acid-induced cholestasis in C57BL/6 J mice	100 mg/kg/day, orally for 7 days	• Protected against cholestasis through PXR activation, ↑ hPXR-regulated genes CYP3A4, UGT1A1, and OATP2 • ↓ Liver necrosis, ALT, AST, and ALP activity • ↓ Serum total bile acids and bilirubin • ↑ Bile acid metabolism, efflux from liver into intestine/feces	[146]
Anti-diabetic	Rat INS-1 pancreatic β-cells and mouse C2C12 skeletal muscle cells	0, 1, 2.5 and 5 μM for 24 h	• Improved hyperglycemia by ↑ insulin secretion, ↑ glucose uptake • ↑ PPARγ, PDX-1, PI3K, Akt, IRS-2 in INS-1 • ↑ IRS-1, AMPK, PI3K, Akt, GLUT-4 in C2C12	[77]
Antiviral	Human monocytic THP-1, PMA-primed, murine fibroblast L929, human embryonic kidney HEK-293 T, peripheral blood mononuclear cells (PBMCs) 10-carboxymethyl-9-acridanone (CMA)-meditated, hepatitis B virus (HBV)-infected C57BL/6 mice	Up to 40 µM, varied durations CMA model: 10 mg/kg, i.p. 1 h before CMA injection, sacrifice after 4 h HBV model: 15 or 30 mg/kg/day, i.p. for 4 weeks	• ↑ cGAS-STING pathway, ↑ IFN-β secretion • ↓ Viral replication • ↑ IFNβ, ISG15, IFIT1, and CXCL10 mRNA	[46]
	HBV-infected C57BL/6 J mice	20 mg/kg, oral gavage, 20 days	• ↓ HBV replication	[45]
*Gomisin A*				
Neuroprotective	Scopolamine-induced cognitive impaired ICR mice	2.5, 5, 10 or 20 mg/kg, orally, 60 min before behavioral tests	• Reversed memory impairment, restored alternation performance, improved spatial learning • ↓ AChE activity	[70]
	LPS-induced N9 microglial cell line (mouse) Primary microglial cells (from newborn Wistar rat cortex) Primary cortical and hippocampal neurons (from embryonic day 18 Wistar rats) Human neuroblastoma SH-SY5Y cells	Up to 100 μM, varied duration	• Protected from neuronal death • ↓ NO and PGE2 production, TNF-α, IL-1β, and IL-6 mRNA and protein, iNOS and COX-2 expressions • ↓ TAK1, IKKα/β, IκB-α phosphorylation, and MAPKs (JNK, ERK1/2, p38), NF-κB nuclear translocation • ↓ Intracellular ROS levels, NADPH oxidase activation, and gp91phox expression	[68]
	Serum and glucose deprivation (SGD) injury in SH-SY5Y cells	2.5–5 µg/ml for 23.5 h	• ↓ NF-κB, IL-1β, and NLRP3 inflammasome activation • ↓ apoptosis, Caspase-1 and Caspase-3, JNK/MAPK signaling	[69]
Anticancer	HepG2, HepG2-DR (P-glycoprotein overexpressing cells)	Up to 100 µM, varied duration	• Restored cytotoxic effects of vinblastine and DOX • Bound to P-gp simultaneously with substrates and altered P-gp-substrate interaction	[90]
	Human colon carcinoma HCT116 carcinoma cells	Up to 40 μM, varied duration	• ↓ Cell proliferation, ↑ apoptosis through caspase-7 activation, ↑ PARP cleavage	[79]
	Human cervical cancer HeLa cells	Up to 100 μM for 72 h	• ↓ Cell proliferation, ↑ cell cycle arrest • ↓ Cyclin D1, pRB, and STAT1	[80]
	CRC cell lines (CT26, MC38, HT29, SW620 BALB/c mice injected with CT26 cells via the tail vein induced lung metastasis	5–100 μM for 24–72 h 50 mg/kg, orally or i.p. for 14 days	• ↓ Lung metastasis via ↑ AMPK/p38 pathway • ↓ Cell viability, migration, invasion, ↑ cell cycle arrest, apoptosis • ↓ Cyclin D1 and CDK4 expression, ↑ cleavage of caspase-9, caspase-3, and PARP, BAX, ↓ BCL-2 and BCL-xL • ↓ EMT via ↑ E-cadherin, ↓ N-cadherin and vimentin • ↓ MMP-2, MMP-9 expression and activity	[87]
	Human melanoma A375 and B16F10 cells C57BL/6 mouse xenograft model with B16F10 melanoma cells	5–50 µM for 24–48 h 10 mg/kg, i.p. for 4 weeks	• ↓ AMPK activation, ↓ ERK/JNK pathway • ↑ Apoptosis, caspase-3 activation, and PARP cleavage • ↓ Migration, invasion, N-cadherin, vimentin, and MMP-9 • ↓ Tumor growth and metastasis in mice	[153]
	Human ovarian cancer SKOV3 and A2780 cells BALB/c-Nu mice with subcutaneous A2780 ovarian cancer xenografts	0.04 µM, varied duration 10 mg/kg, i.p., every 2 days for 14 days	• ↑ Paclitaxel (PTX)'s effectiveness • ↑ Cell cycle arrest without promoting apoptosis, ↓ ROS production • ↓ MMP-2, CDK4 and cyclin B1, PI3K/Akt and Keap-1/Nrf2 pathways • ↓ Tumor growth without significant toxicity in mice	[125]
Vasorelaxant	Isolated rat thoracic aorta rings	1 µM to 100 µM, varied duration	• ↑ Endothelium-dependent and direct vascular relaxation via NO, prostacyclin, and endothelium-derived hyperpolarizing factor (EDHF), ↓ MLC phosphorylation	[154]
	Human coronary artery endothelial cells	10 µM, varied duration	• ↑ Endothelial-dependent vasorelaxation via Ca^2^⁺-dependent activation and translocation of endothelial nitric oxide synthase (eNOS), ↑ NO production	[155]
	Angiotensin II (Ang II)-induced hypertension in C57BL/6 mice Human coronary artery endothelial cells, human umbilical vein endothelial cells Vascular smooth muscle cells (VSMCs) (Mouse aorta-derived)	2 or 10 μg/kg/min, continuously administered subcutaneously via an osmotic minipump for 14 days 1 to 10 μM for 24 h before Ang II exposure	• ↓ Ang II-induced hypertension, blood pressure • ↓ ROS, NO production • ↑ Phosphorylation of eNOS at Ser1177	[156]
Hepatoprotective	CCl4-induced hepatic and renal injury in SD rats	100 mg/kg/day, oral gavage, 4 days pre-treatment before CCl4 administration	• ↓ CCl4-induced liver and kidney damage, ↓ hepatocellular necrosis, less central vein dilation in liver, and improved glomerular structure and convoluted tubules in kidney • ↓ Serum biochemical markers (ALP, AST, ALT for liver toxicity; BUN, creatinine for kidney toxicity) • ↓ Caspase-3 activation • ↑ ERK and p38 phosphorylation in liver • ↑ p38 and JNK phosphorylation, ↓ ERK phosphorylation in kidney	[157]
	Acetaminophen (APAP)-induced liver injury in C57BL/6 mice	200 mg/kg/day, orally, seven doses at 12-h intervals before APAP treatment	• ↓ APAP-induced liver damage, and ALT/AST levels • ↑ Total and mitochondrial GSH • ↓ CYP2E1, CYP1A2, and CYP3A11 activity, NAPQI-GSH formation	[51]
	Cholestatic liver injury in C57BL/6 J mice	100 mg/kg, orally, twice daily for 7 days	• ↓ Liver necrosis, ALT, AST, ALP, TBA, and Tbil levels and mortality • ↓ Pyroptosis, LDH release, TUNEL-positive hepatocytes, and pore formation in cell membranes • ↓ NLRP3, ASC, caspase-1, and GSDMD and Apaf-1, caspase-11 p20, caspase-3 p20, and GSDME • ↓ NF-κB activation, ↓ IL-6, TNF-α, iNOS, COX-2, ICAM-1, and MCP-1 • ↓ FoxO1, CDKN1A, CDKN1B, and RBL2 • ↑ PXR activation, CYP2B10 and CYP3A11 expression	[19]
	Lithocholic acid-induced cholestasis in C57BL/6 J mice	100 mg/kg/day, orally for 7 days	• Protected against cholestasis through PXR activation, ↑ hPXR-regulated genes CYP3A4, UGT1A1, and OATP2 • ↓ Liver necrosis, ALT, AST, and ALP activity • ↓ Serum total bile acids and bilirubin • ↑ Bile acid metabolism, efflux from liver into intestine/feces	[146]
Antioxidant	MC3T3-E1 pre-osteoblast cells (mouse calvaria-derived cell line)	1 and 10 µM, varied duration	• ↑ Osteoblast differentiation and mineralization, mitochondrial biogenesis • ↑ BMP-2, BMP-7, OPG, Runx-2 expression, and alkaline phosphatase (ALP) activity • ↑ HO-1, PGC-1α, NRF-1, and TFAM expression • ↓ ROS levels in high glucose condition, ↑ Cu/Zn-SOD and Mn-SOD	[158]
*Gomisin B*				
Neuroprotective	Depressive Kunming mice	10, 15 and 20 mg/kg/day, i.p. for 10 days	• Cognition improvement • ↑ GLT-1 levels via PI3K/Akt/mTOR pathway	[159]
	Lithocholic acid-induced cholestasis in C57BL/6 J mice	100 mg/kg/day, orally for 7 days	• Protected against cholestasis through PXR activation, ↑ hPXR-regulated genes CYP3A4, UGT1A1, and OATP2 • ↓ Liver necrosis, ALT, AST, and ALP activity • ↓ Serum total bile acids and bilirubin • ↑ Bile acid metabolism, efflux from liver into intestine/feces	[146]
	APP/PS1 AD mice	20 mg/kg/d, orally, 6 weeks	• Improved cognitive dysfunction • Enhanced effect with Osthole • ↓ β-site APP-Cleaving Enzyme 1 (BACE1), ↓ oxidative stress, anti-apoptotic	[160]
*Gomisin C*				
Neuroprotective	Serum and glucose deprivation (SGD) injury in SH-SY5Y cells	2.5–5 µg/ml for 23.5 h	• ↓ NF-κB, IL-1β, and NLRP3 inflammasome activation • ↓ Apoptosis, caspase-1 and caspase-3, JNK/MAPK signaling	[69]
Hepatoprotective	Liver ischemia–reperfusion (I/R) injury in C57BL/6 mice	0, 25, 50, 100, 200, 400, and 800 mg/kg/day, oral gavage, 5 days before the induction of I/R injury	• ↓ Oxidative/nitrosative stress, ↓ MDA, NO, ↑ GSH/GSSG, ATP levels • ↓ Serum levels of IL-1β, IL-6, and TNF-α, neutrophil infiltration • ↓ Apoptosis, ↑ BCL-2, ↓ BAX, cleaved caspase-3 • ↓ JNK, p38 and ERK MAPK pathway	[52]
	Lithocholic acid-induced cholestasis in C57BL/6 J mice	100 mg/kg/day, orally for 7 days	• Protected against cholestasis through PXR activation, ↑ hPXR-regulated genes CYP3A4, UGT1A1, and OATP2 • ↓ Liver necrosis, ALT, AST, and ALP activity • ↓ Serum total bile acids and bilirubin • ↑ Bile acid metabolism, efflux from liver into intestine/feces	[146]
*Gomisin G*				
Anticancer	TNBC MDA-MB-231 and MDA-MB-468 cells	0, 1, 5, and 10 µM for 3 and 5 days	• ↓ Proliferation rather than apoptosis, ↑ cell cycle arrest, ↓ cyclin D1 • ↓ Akt phosphorylation, ↓ retinoblastoma tumor suppressor protein (Rb) and phosphorylated Rb	[161]
	Human colon cancer LoVo cells	0, 1, 5, and 10 µM for 3 and 5 days	• ↓ Viability and colony formation, ↑ apoptosis, ↑ cell cycle arrest • ↑ Cleaved poly-ADP ribose polymerase (PARP) and caspase-3 proteins, ↓ phosphorylated Rb protein • ↓ Akt phosphorylation, PI3K/Akt pathway	[85]
*Gomisin J*				
Anti-inflammatory	LPS-stimulated murine macrophage RAW 264.7 cells	1–10 µM for 24 h	• ↓ TNF-α, IL-6, and IL-1β mRNA and protein • ↓ NO secretion • ↓ p38 MAPK, ERK1/2, JNK phosphorylation	[151]
Hepatoprotective	Oleic acid-induced hepatic lipogenesis in human liver cancer HepG2 cells	2.5, 5 or 10 μM for 24 h	• ↓ Hepatic steatosis, lipogenesis, ↑ fatty acid oxidation, ↓ intracellular lipid accumulation • ↑ pAMP, ↓ acetyl-CoA carboxylase (ACC), fatty acid synthase (FAS), and fetuin-A	[162]
Anticancer	Human Colon Cancer Cells: HCT116	5–20 μM, 24–72 h	• ↑ cell cycle arrest without apoptosis • ↓ Wnt/β-catenin signaling by preventing β-catenin from binding to DNA at TCF binding sites • ↓ Cyclin D1, Cdk2, and Cdk4	[150]
	Human breast cancer MCF7, MDA-MB-231 cells, 13 different cancer cell lines, including colon (HCT116, HT-29) and cervical (HeLa, SiHa)	1 to 30 µg/ml for 24, 48, and 72 h	• ↓ Cell viability in MCF7 and MDA-MB-231 • ↑ Apoptosis and necroptosis	[83]
	Human glioma cell lines U87 and U251 Glioma (U251) xenograft mouse model (BALB/C nude mice)	20, 40, and 60 μM for 24 h 40 mg/kg for 4 weeks	• ↑ Mitochondrial-regulated apoptosis, ↑ cleaved Caspase-3, Caspase-8, and BAX, cytochrome c (Cyt-c) release from mitochondria to cytoplasm, ↓ BCL-2 • ↑ Oxidative stress, ↓ aerobic glycolysis (↓ glucose uptake and lactate production, ↓ ATP levels, ↑ oxygen consumption rate) • ↓ Hexokinase II (HKII) expression and HKII binding to voltage-dependent anion channel (VDAC) in mitochondria • Tumor suppression with minimal toxicity in mice, ↓ Ki-67 protein	[163]
Neuroprotective	Cerebral ischemia/reperfusion injury in Wistar rats	5, 10, 20, 40, and 80 mg/kg, i.p., before cerebral reperfusion	• ↓ Neurological deficits, cerebral infarction size, and brain water content • Improved neuronal survival in hippocampus • ↓ Cleaved caspase-3 and BAX, ↑ BCL-XL, ↓ TUNEL-positive apoptotic cells in ischemic brain tissue • ↓ NF-κB activation (p-p65) and COX-2 expression, NO levels • ↑ Nrf2 nuclear translocation and HO-1 expression, SOD, GSH, GSH-px activities • ↓ MDA	[164]
Anti-inflammatory				
Antioxidant				
*Gomisin K3*				
Antioxidant	WT or ChAT-HEK 293 cells	10–100 µM, 30 min prior to t-butyl hydroperoxide (tBHP) treatment	• ↓ Intracellular oxidative stress and cytotoxicity induced by tBHP	[165]
	Preimplantation porcine embryos	0, 10, 20, and 50 μM for 7 days	• ↑ Blastocyst formation rate, ↑ proliferation, ↓ apoptosis • ↓ ROS, ↑ GSH and esterase activity • ↑ Mitochondrial membrane potential, ATP production • ↓ ERK1/2, JNK1/2/3, and p38 MAPK phosphorylation	[166]
Cardioprotective	Human umbilical vein endothelial cells	10 or 20 μM 2 h before oxidized low-density lipoprotein (OxLDL) exposure	• ↓ Endothelial dysfunction, cell shrinkage, membrane blebbing, improved cell viability and membrane integrity • ↓ LOX-1 expression • Restored eNOS phosphorylation and ↓ iNOS activation • ↓ p38 MAPK phosphorylation, NF-κB activation • ↓ IL-8 and COX-2 levels, ↓ ICAM-1, VCAM-1, and E-selectin	[167]
Anti-inflammatory				
Antioxidant				
Neuroprotective	1-Methyl-4-phenylpyridinium iodide (MPP +)-induced neurotoxicity in human neuroblastoma SH-SY5Y cells	1 μM, 10 μM, and 50 μM for 24 h before MPP + exposure	• ↑ Cell viability, ↓ apoptosis • ↓ DNA fragmentation, ↓ caspase-3 activity • ↑ BCL-2/BAX ratio, ↑ TRX1 mRNA and protein • ↓ ASK1 and p38 phosphorylation, and NF-κB translocation	[168]

### Antioxidant activity

Oxidative stress arises from a disruption in the balance between the pro-oxidant and antioxidant systems, leading to an overproduction of reactive oxygen species (ROS). These molecules are primarily formed in mitochondria during respiration, but additional sources include redox metal ions and enzymatic reactions [24]. To counteract ROS, the body relies on enzymatic antioxidants such as superoxide dismutase (SOD), catalase (CAT), glutathione peroxidase (GPx) and glutathione reductase (GR), alongside nonenzymatic antioxidants such as vitamins C and E and reduced glutathione (GSH) [25].

Among natural antioxidant sources, *Schisandra chinensis* fruit extract has garnered attention for its potent ROS-scavenging properties. The major constituents of dibenzocyclooctadiene lignans are considered to be responsible for the antioxidant activities of *Schisandra chinensis* fruit extract [9, 26]. Notably, the ROS scavenging activity of schisandrin B is comparable to that of vitamin C [27].

Beyond their ROS-scavenging ability, *Schisandra* lignans play a vital role in oxidative stress regulation by directly scavenging ROS, enhancing enzymatic antioxidant defenses such as those of SOD and GPx under normal conditions, and acting as pro-oxidants in pathological states. For example, schisandrin B and deoxyschisandrin possess strong antioxidant properties by enhancing enzymatic activities such as those of SOD and GPx, maintaining GSH levels, and reducing the levels of oxidative stress markers such as malondialdehyde (MDA) and ROS [7]. These effects have been demonstrated in various models of neurotoxicity and oxidative stress, including D-galactose-induced cognitive deficits [28], scopolamine- and cisplatin-induced cerebral oxidative damage [29] and amyloid-beta-induced memory impairment [30].

Additionally, *Schisandra* lignans exert protective effects beyond neurodegenerative models. For example, schisandrin B has been shown to mitigate oxidative stress-associated skin damage. Gao et al*.* reported that schisandrin B exhibited potent antioxidant activity by blocking the cyclooxygenase-2 (COX-2), interleukin-6 (IL-6) and interleukin-18 (IL-18) signaling pathways, thereby inhibiting collagen degradation through the suppression of matrix metalloproteinase (MMP) expression, offering protection against ultraviolet B (UVB)-induced skin damage and ROS [31].

### Anti-inflammatory activity

The anti-inflammatory effects of *Schisandra chinensis* lignans are primarily mediated through the inhibition of proinflammatory signaling pathways, including inducible nitric oxide synthase (iNOS) and COX-2, Nuclear Factor kappa-light-chain-enhancer of activated B cells (NF-κB) and mitogen-activated protein kinases (MAPK), which lead to a reduction in proinflammatory mediators [nitric oxide (NO) and prostaglandin E2 (PGE-2)] and proinflammatory cytokines [tumor necrosis factor alpha (TNF-α), IL-6, interferon gamma (INF-γ), interleukin-1β (IL-1β) and monocyte chemoattractant protein-1 (MCP-1)] [8]. *Schisandra chinensis* lignans suppressed the phosphorylation of key signaling molecules, including IκB kinase alpha/beta (IKKα/β), inhibitor of nuclear factor kappa-B alpha (IκBα), protein kinase B (Akt), TANK-binding kinase 1 (TBK1), ERK, p38, JNK, NF-κB (p65), c-Jun, and interferon regulatory factor 3 (IRF3), thereby disrupting the IKKα/β/NF-κB, MAPK/AP-1 (c-Jun) and TBK1/IRF3 pathways, which play pivotal roles in inflammation [32].

Schisandrin A has been extensively studied for its anti-inflammatory effects. Schisandrin A significantly suppressed lipopolysaccharide (LPS)-induced production of proinflammatory mediators, such as NO and PGE2, by reducing the expression of iNOS and COX-2 at both the mRNA and protein levels in RAW 264.7 macrophages. Additionally, Schisandrin A inhibited the secretion of proinflammatory cytokines, including TNF-α and IL-1β, by downregulating their gene and protein expression. It further blocked the LPS-induced nuclear translocation of NF-κB and the activation of MAPK and PI3K/Akt signaling. In addition, schisandrin A reduced LPS-stimulated ROS accumulation while increasing the expression of antioxidant factors, such as nuclear factor erythroid 2-related factor 2 (NRF2) and heme oxygenase-1 (HO-1) [33]. In IL-1β-stimulated human osteoarthritis chondrocytes, schisandrin A reduced NO production and suppressed iNOS and COX-2 activities, effectively blocking the NF-κB and MAPK signaling pathways and thereby mitigating inflammation [34]. Similarly, in mouse models of LPS-induced mastitis, schisandrin A alleviated mammary injury by suppressing proinflammatory mediators and activating the NRF2 signaling pathway. The critical role of NRF2 was confirmed, as these effects were partially reversed by NRF2 inhibitors. Furthermore, schisandrin A counteracts LPS-induced suppression of autophagy by activating AMP-activated protein kinase- Unc-51-like autophagy activating kinase 1 (AMPK-ULK1) signaling and inhibiting mammalian target of rapamycin (mTOR) phosphorylation, contributing to its anti-inflammatory effects [35].

Schisandrin B has also demonstrated robust anti-inflammatory effects by modulating multiple pathways. It inhibited NF-κB/MAPK [36], Toll-like receptor 4 (TLR4)-dependent MyD88/IKK [37] and phosphorylated (p)-53 [38]. Additionally, it activated the peroxisome proliferator-activated receptor-gamma (PPAR-γ) and NRF2 pathways while suppressing NF-κB activity [39–41].

Together, schisandrin lignans such as schisandrin A and B demonstrate broad anti-inflammatory activity through their ability to inhibit pro-inflammatory mediators, suppress critical signaling pathways such as NF-κB and MAPKs, and enhance antioxidant defenses via NRF2 activation. These properties underscore the therapeutic potential of *Schisandra chinensis* lignans in managing inflammation-related conditions, including arthritis, osteoarthritis, mastitis, and other inflammatory diseases.

### Antimicrobial/viral activity

*Schisandra chinensis* extracts have demonstrated significant antibacterial activity against both gram-positive and gram-negative bacteria, with gram-positive bacteria showing greater sensitivity. This heightened susceptibility is attributed to structural differences in the bacterial cell walls, as gram-positive bacteria lack the outer membrane that protects gram-negative bacteria, increasing their vulnerability to the bioactive compounds in *Schisandra chinensis* [9]. Bai et al*.* investigated the activity of *Schisandra chinensis* fruit ethanolic and water extracts against common food-borne pathogens and food-spoiling organisms. Both extracts displayed strong antibacterial activity against *Staphylococcus aureus*, *Listeria monocytogenes*, *Bacillus subtilis*, *B. cereus*, *Salmonella enterica* subsp. *enterica* serovar Typhimurium, *Pseudomonas aeruginosa*, *Enterobacter aerogenes* and *Escherichia coli* [42]. Interestingly, antimicrobial assays revealed that *Schisandra chinensis* leaf extracts were even more effective than fruit extracts against targeted bacteria, underscoring the importance of plant part selection in maximizing antibacterial efficacy [9].

In addition to its antibacterial properties, *Schisandra chinensis* also has antiviral activity. A study by Xu et al*.* revealed that schisandrin B and deoxyschizandrin effectively inhibited human immunodeficiency virus 1 (HIV-1) reverse transcriptase-associated DNA polymerase activity, which is essential for viral replication. Notably, schisandrin B effectively targeted HIV-1 reverse transcriptase mutants linked to drug resistance and interfered with the early stages of viral replication [43]. In another study, schisandrin A was found to suppress dengue virus replication by increasing antiviral interferon responses via the signal transducer and activator of transcription (STAT)1/2 signaling pathway [44]. More recently, schisandrin C has been shown to activate the cGAS-STING pathway and promote interferon beta (IFN-β) production in THP-1 macrophages, leading to the inhibition of hepatitis B virus (HBV) replication in HepG2.2.15 cells. Additionally, the combination of schisandrin C and luteolin has a synergistic effect on the inhibition of HBV replication in HBV-infected mice, suggesting a promising therapeutic strategy [45, 46].

These findings highlight the antimicrobial and antiviral properties of *Schisandra chinensis*, making it a compelling candidate as a natural therapeutic agent for managing bacterial and viral infections. Future research should focus on exploring the synergistic potential of *Schisandra* with existing antimicrobial or antiviral agents to increase its clinical utility and efficacy. Additionally, further studies are needed to evaluate its mechanisms of action, optimize extraction methods for different bioactive components, and assess its efficacy in human clinical trials to establish its role in combating infectious diseases.

### Hepatoprotective effect

Liver protection is one of the most prominent pharmacological effects of *Schisandra chinensis*, as its fruits and extracts are widely used in China to manage chemical and viral liver injuries [47]. This protective effect is mediated through multiple mechanisms, including enhanced liver detoxification and tissue regeneration. A key mechanism involves the modulation of cytochrome P450 enzymes, such as CYP3A and CYP2E1, which play critical roles in xenobiotic metabolism. By increasing the activity of these enzymes, *Schisandra chinensis* improved the hepatic clearance of toxins and drugs, thus facilitating detoxification processes [48]. Furthermore, treatment with *Schisandra chinensis* has been shown to increase the intracellular level of GSH and stimulate its synthesis in hepatocytes. As a major intracellular antioxidant, GSH detoxifies free radicals and reactive metabolites, providing protection against oxidative damage to liver cells.

Schisandrin B exemplified the hepatoprotective effects of *Schisandra chinensis*. It promoted hepatic differentiation of human umbilical cord mesenchymal stem cells via activation of the Janus kinase 2 (JAK2)/signal transducer and activator of transcription 3 (STAT3) pathway, which in turn alleviated liver fibrosis in mouse models [49]. Schisandrin B also protected against carbon tetrachloride (CCl₄)-induced hepatotoxicity by increasing mitochondrial GSH levels and increasing the activity of key antioxidant enzymes such as GR, GPx, and glutathione S-transferases. Moreover, it increased hepatic levels of vitamins C and E, further strengthening the liver's antioxidant defenses [50].

Gomisin A, another active lignan, increased the microsomal activity of cytochrome B5, cytochrome P450, nicotinamide adenine dinucleotide phosphate (NADPH) cytochrome C reductase, N-demethylase and 7-ethoxycoumarin O-deethylase while reducing the activity of 3,4-dibenzopyrene hydroxylase. It also promoted hepatocyte proliferation, enhanced endoplasmic reticulum function and improved hepatic blood flow [2]. Additionally, gomisin A has been linked to the activation of PXR, which accelerated bile acid metabolism, promoted bile acid efflux and induced hepatic expression of CYP3A, further supporting its hepatoprotective effects [17, 19].

A further study on the hepatoprotective effects of six *Schisandra* lignans, namely, gomisin A, schisandrin, deoxyschisandrin (schisandrin A), schisandrin B, schisandrin C and schisantherin A, on acetaminophen-induced liver injury revealed that these effects are partially associated with the inhibition of cytochrome-mediated bioactivation. The results of the morphological and biochemical assessments in this study demonstrated the protective effects of all the tested lignans against acetaminophen-induced liver injury [51]. Further evidence supported the role of schisantherin A in protecting against liver ischemia‒reperfusion injury and alcohol-induced liver damage. Its protective effects were found to be linked to its ability to modulate oxidative stress and inflammatory responses in the liver [52, 53].

Collectively, these findings highlight the multifaceted hepatoprotective mechanisms of *Schisandra chinensis*, which include the modulation of cytochrome P450 enzymes, the enhancement of antioxidant defenses, the stimulation of tissue regeneration and the reduction in inflammatory responses. These attributes make *Schisandra chinensis* a promising natural therapeutic agent for managing various liver diseases.

### Neuroprotective effects

The neuroprotective properties of *Schisandra chinensis* lignans have garnered significant attention for their potential to combat neurodegenerative disorders such as Alzheimer’s disease (AD), Parkinson’s disease (PD), Huntington’s disease and stroke. These effects are primarily linked to their ability to reduce oxidative stress, modulate neuroinflammation and improve cognitive function in various cellular and animal models [3, 54]. Key lignans, including schisandrin A, B and C, as well as gomisin A and N, have demonstrated substantial efficacy in these roles.

Schisandrin has significant antioxidant and anti-inflammatory properties, particularly in PD models, where it reduced oxidative stress and inflammation [55]. It has been shown to mitigate dexamethasone-induced neurotoxicity, improve cell viability in cerebral cortical cells and restore cognitive performance and histological integrity in mice [56]. In models of amyloid-β (Aβ1)–42-induced memory impairment, schisandrin enhanced cognitive function by improving short-term and spatial memory while increasing the levels of antioxidant enzymes (SOD and GPx) and GSH and reducing the level of MDA in the brain [57]. In a model of scopolamine-induced memory impairment, schisandrin improved cognitive performance by reversing passive avoidance deficits [58]. In ovariectomy-induced rats, it enhanced cognitive function by lowering acetylcholinesterase (AChE) activity and malondialdehyde (MDA) levels while increasing SOD activity and NADPH-diaphorase-positive neurons [59].

Among *Schisandra chinensis* lignans, schisandrin B is considered the most effective neuroprotective agent. It mitigated oxidative stress by reducing MDA and ROS while increasing SOD activity and GSH production, effectively alleviating anxiety-like behaviors in mice [60]. Schisandrin B also ameliorated Aβ-induced oxidative stress and neuronal dysfunction in the cortex and protected primary cortical neurons from Aβ1–42 toxicity by regulating apoptotic pathways, including the upregulation of BCL-2 and the suppression of BAX, cytochrome c release and caspase activation [61, 62]. In vitro studies further revealed that schisandrin B suppressed oxidative stress and inflammation in BV2 microglia and PC12 cells [63, 64] and protected against microglial-mediated inflammatory damage in microglia–neuron cocultures by downregulating NADPH oxidase and other mediators, such as NO, TNF-α, PGE-2, IL-1β and IL-6 [37]. Additionally, schisandrin B mitigated ischemic brain injury in rats by suppressing the expression of the inflammatory proteins TNF-α and IL-1β and degrading the matrix metallopeptidases MMP-2 and MMP-9 in ischemic hemispheres [65]. Mechanistically, the anti-inflammatory effects of schisandrin B are linked to the inhibition of the TLR4-mediated MyD88/IKK/NF-κB signaling pathways [37].

Although less extensively studied, schisandrin C has demonstrated potent neuroprotective and anti-neuroinflammatory effects. It significantly improved cognitive performance in Aβ1-42-induced AD model mice and increased the activity of antioxidant enzymes (SOD and GPx) while reducing cholinesterase activity in the hippocampus and cortex [66]. Schisandrin C also inhibited lipoteichoic acid-induced inflammation by reducing the levels of proinflammatory mediators (PGE-2, NO and ROS) and suppressing NF-κB, JAK/STAT and MAPK activation [67].

Gomisin A has demonstrated significant neuroprotective effects, particularly in preventing neuroinflammation in LPS-stimulated N9 microglia. It inhibited NO, PGE-2 and proinflammatory cytokines (TNF-α, IL-1β and IL-6) while reducing ROS production and NADPH oxidase activation. Gomisin A also protected SH-SY5Y cells and rat cortical neurons from damage caused by activated microglia through TLR4-mediated NF-κB and MAPK signaling inhibition [68]. Furthermore, it improved cognitive performance in scopolamine-induced memory impairment models and protected against serum and glucose deprivation injury in neuronal cells [69, 70]. In summary, *Schisandra chinensis* lignans exhibit a broad spectrum of neuroprotective effects by targeting oxidative stress, neuroinflammation and neuronal death, making them promising candidates for the management of neurodegenerative disorders. However, further research is necessary to establish their clinical efficacy and bioavailability.

### Antidiabetic effect

Lignans derived from *Schisandra chinensis* have shown significant potential in regulating glucose metabolism and enhancing insulin sensitivity, making them promising candidates for managing type 2 diabetes (T2D) [71, 72]. Among these, schisandrin B has been found to stimulate insulin secretion both in vivo and in vitro via the glucagon-like peptide-1 receptor/cyclic AMP/protein kinase A (GLP-1R/cAMP/PKA) signaling pathway [73]. Schisandrin B (Gomisin N) has substantial metabolic benefits through the activation of AMPK/Akt signaling in C2C12 myotubes, leading to increased glucose uptake, mitochondrial biogenesis and fatty acid oxidation. In high-fat diet (HFD)-induced obese mice, gomisin N effectively reduced hyperglycemia and improved glucose tolerance, underscoring its therapeutic potential for metabolic disorders such as obesity and T2D [74]. Schisandrin A has shown protective effects against oxidative stress and inflammation in a diabetic nephropathy (DN) mouse model. It also reduced ferroptosis induced by high glucose levels and pyroptosis caused by ROS, further demonstrating its potential for alleviating diabetic complications [75]. Similarly, schisandrin mitigated DN induced by streptozotocin and a high-fat diet (STZ/HFD) in mice, primarily by inhibiting transforming growth factor beta 1 (TGF-β1)-mediated renal fibrosis and reducing inflammation via modulation of the PI3K/Akt and NF-κB signaling pathways [76]. Additionally, schisandrol A and schisandrin C enhanced insulin secretion in response to high glucose levels without exerting toxic effects on rat INS-1 pancreatic β-cells. Notably, schisandrin C improved hyperglycemia by increasing insulin secretion in pancreatic β-cells and facilitating glucose uptake into skeletal muscle cells. Its effects are superior to those of gliclazide, a commonly prescribed drug for T2D management [77]. Collectively, these findings highlight the potential of *Schisandra chinensis* lignans as multifunctional agents for the treatment of T2D and its associated complications.

### Anticancer activity

One of the most significant pharmacological properties of *Schisandra chinensis* lignans is their anticancer activity. Lignans such as schisandrin A, schisandrin B, schisandrin, schisandrol B, gomisin M2, gomisin G and schisantherin A (gomisin C) have promising anticancer effects [11]. These effects are mediated through multiple mechanisms, including the induction of apoptosis, the inhibition of cell proliferation, the suppression of metastasis and the prevention of angiogenesis [78].

For example, gomisin A induced apoptosis in colon carcinoma HCT-116 cells via caspase-7 activation [79] and inhibited cell proliferation via cell cycle arrest in HeLa cells exposed to TNF-α [80]. Gomisin N (Schisandrin B) exerts anticancer effects by promoting cell death and inhibiting proliferation at high concentrations [81]. Similarly, deoxyschisandrin selectively induced apoptosis in colon adenocarcinoma cells, whereas gomisin N induced cell death in both colon and ovarian adenocarcinoma cells [82]. In a study by Jung et al*.* Gomisin J was shown to suppress proliferation and induce necroptosis in the breast cancer cell lines MCF7 and MDA-MB-231, particularly in apoptosis-resistant cancers, making it a potential therapeutic for hard-to-treat malignancies [83]. Recently, our laboratory showed that schisandrin B suppressed colon cancer growth by inducing cell cycle arrest and apoptosis in vitro and in vivo [84].

*Schisandra chinensis* lignans also target key signaling pathways involved in cancer progression. For example, gomisin G reduced Akt phosphorylation and modulated the PI3K/Akt pathway, leading to apoptosis in colorectal cancer (CRC) cells [85]. Schisandrin A, an inhibitor of heat shock factor 1 (HSF1) in CRC cell lines, induced cell cycle arrest and apoptosis, targeting a critical pathway in cancer cell survival [86]. Furthermore, gomisin A halted cell cycle progression in CRC cells by inducing G0/G1 phase arrest and activating the AMPK and p38 pathways, leading to apoptosis [87]. Schisantherin A induced apoptosis in gastric cancer cells via JNK activation, which was linked to a decrease in NRF2 levels [88]. Additionally, schisandrin B inhibited STAT3 phosphorylation and nuclear translocation in triple-negative breast cancer (TNBC), resulting in reduced tumor migration in mice [89].

In addition to these direct anticancer mechanisms, lignans such as schisandrin B and gomisin A have been shown to reverse MDR by inhibiting P-gp, a drug efflux transporter associated with reduced chemotherapy efficacy [90, 91]. Schisandrin B enhanced the cytotoxic effects of drugs such as doxorubicin (DOX), improving their therapeutic potential [91–94]. It also inhibited TNBC cell growth and migration by suppressing STAT3-mediated signaling and epithelial-to-mesenchymal transition (EMT) [89, 95]. Other lignans, including gomisin A, gomisin C, schisandrin and schisandrin A, have similarly been shown to reverse P-gp-mediated MDR and reduce drug efflux in cancer cells [90, 96–98].

The diverse anticancer properties of *Schisandra chinensis* lignans, ranging from the inhibition of cell proliferation and migration to the induction of apoptosis and overcoming drug resistance, highlight their potential as therapeutic agents in cancer treatment. Combining these lignans with existing therapies offers a promising strategy to increase treatment efficacy and overcome drug resistance, warranting further investigation and clinical exploration.

## Challenges and limitations of the use of *Schisandra chinensis* and its lignans

Despite the significant therapeutic potential of *Schisandra chinensis* and its lignans, several challenges and limitations are associated with their use, particularly in clinical settings. These issues primarily stem from poor bioavailability, variability in extraction and quantification methods, uncertainties regarding long-term safety, and a lack of robust clinical evidence from human trials.

### Poor bioavailability

One of the primary issues is the poor bioavailability of these compounds. Lignans such as schisandrin and gomisin A are highly lipophilic, which limits their solubility in water, making their absorption in the GI tract inefficient [99]. After oral administration, these compounds often undergo extensive first-pass metabolism in the liver, where they are rapidly metabolized before reaching systemic circulation. This leads to reduced therapeutic concentrations in the body, limiting their efficacy [99].

### Variability in extraction and quantification methods

Another significant challenge is the inconsistent composition of bioactive compounds in *Schisandra chinensis* extracts, which can vary depending on factors such as geographical location, harvest time and processing methods [1, 9, 100].

Recent studies have explored various extraction methods to optimize lignan yield and consistency. For instance, a study by ref. [101] developed a diol-based matrix solid-phase dispersion (MSPD) method combined with high-performance liquid chromatography (HPLC) for the simultaneous extraction and determination of ten lignans from *Schisandra chinensis*. This method allowed for efficient extraction and quantification, facilitating quality control across different samples. Another approach, known as smashing tissue extraction (STE), was optimized by researchers to extract five lignans from *Schisandra chinensis* fruits. This technique proved to be more effective and less time-consuming compared to classical methods, offering a promising alternative for lignan extraction [102].

A comprehensive review highlighted the variability in lignan content among different *Schisandra* species [12], further complicating standardization in therapeutic formulations. To address this, researchers have developed advanced analytical methodologies, including ultra-performance liquid chromatography (UPLC) [103–106] and ultra-fast liquid chromatography (UFLC) [107], which provide high-precision quantification of lignans. Additionally, novel extraction and purification techniques continue to be refined to improve efficiency and reproducibility. Traditional methods such as hot water extraction remain widely used due to their simplicity, cost-effectiveness, and operational feasibility [108]. However, more advanced techniques have emerged, offering greater efficiency and selectivity. Microwave-assisted extraction (MAE) has been explored as a method to improve extraction efficiency while reducing processing time [109, 110]. Meanwhile, enzymatic hydrolysis has gained attention for its potential to shorten extraction time, lower solvent consumption, and improve the quality of extracted compounds [111]. Flash extraction has been introduced as a technique to accelerate compound separation and purification [112], while the aqueous two-phase system extraction offers a sustainable and selective separation process [113, 114]. More recently, an enzymatic pretreatment combined with ionic liquid and ultrasonic-assisted extraction (EIPU) technique was successfully developed for the efficient extraction of lignans from the anthocyanin residue of *Schisandra chinensis* [111]. Overall, the continued refinement of extraction and purification methods is crucial to achieving consistent lignan composition and concentrations, ensuring reproducibility, quality and efficacy in therapeutic applications.

### Potential adverse effects of long-term use in humans

While *Schisandra chinensis* is generally considered safe at moderate doses [12, 115], data on its long-term safety, particularly with concentrated extracts or lignan-rich supplements, remains limited. Short-term use appears to be well-tolerated, but prolonged consumption—particularly at high doses—raises concerns about potential adverse effects. The lack of comprehensive toxicological and pharmacokinetic studies hampers its widespread adoption in clinical settings. Further research is needed to establish safety profiles, optimal dosages, and potential interactions with other medications.

Given its growing use in complementary and alternative medicine, a thorough evaluation of its safety profile is particularly critical in the context of polypharmacy. *Schisandra chinensis* has been reported to interact with multiple drug classes, posing clinical implications for co-administration with conventional therapies. A key challenge lies in its interaction with drug-metabolizing enzymes, particularly cytochrome P450 enzymes, particularly CYP3A4, which is involved in the metabolism of many drugs [12, 116, 117]. This interaction can lead to potential drug‒drug interactions, where the concurrent use of *Schisandra chinensis* with other medications might either inhibit or induce the metabolism of those drugs, causing either toxicity or reduced efficacy [12].

Several studies highlight the pharmacokinetic impact of other *Schisandra* species on drug metabolism. In a rat model, co-administration of Wuzhi tablets—an ethanol extract of *Schisandra sphenanthera* containing schisandrin, schizandrol B, schisantherin A, schisanhenol, and deoxyschizandrin—significantly altered cyclosporine A blood levels, suggesting sensitivity to CYP3A/P-gp modulation [118, 119], Wuzhi capsules found that Wuzhi tablets influenced cyclophosphamide metabolism, potentially mitigating its nephrotoxic and hepatotoxic effects. Additionally, Wuzhi tablets slightly increased tacrolimus blood concentration, while *Schisandra* fruit extract enhanced tacrolimus absorption by modulating first-pass metabolism in the gut and liver [120]. These findings underscore the complexity of *Schisandra-*drug interactions, particularly for patients on long-term pharmacological treatments such as immunosuppressants and chemotherapeutics.

Beyond pharmacokinetics, the therapeutic potential of *Schisandra chinensis* lignans in oncology remains under investigation. A key challenge is their specificity and selectivity toward cancer cells versus normal cells, as well as the potential for prolonged treatment to induce drug resistance in cancer populations [78]. Understanding the extent of potential drug-drug interactions is crucial for ensuring patient safety, particularly in those requiring complex medication regimens. Future clinical trials and pharmacokinetic assessments are needed to establish standardized guidelines for its safe and effective use alongside conventional therapies.

### Lack of clinical evidence and human trial data

While the mechanisms of action of *Schisandra chinensis* and its lignans are well-documented, the majority of research is preclinical, with only a few human studies available [121–124]. Although some preliminary clinical trials suggest beneficial effects in chronic liver disease, fatigue syndromes, neurological/ psychiatric disorders and infectious diseases, the sample sizes are often small, and the study designs vary significantly [2, 121]. Without well-controlled, large-scale randomized clinical trials (RCTs), it remains difficult to establish standardized dosages, pharmacokinetics and long-term safety.

Certain *Schisandra* lignans have also been investigated as a chemosensitizing agent, particularly for its ability to reverse MDR [86, 90]. Preclinical studies indicate that lignans can enhance the efficacy of chemotherapeutic agents such as paclitaxel and DOX [93, 125]. However, translating these findings into clinical applications remains complex due to challenges related to bioavailability, tumor microenvironment interactions, and genetic variability among patients. These factors may significantly influence the efficacy of lignans as MDR modulators, necessitating further investigation in well-designed clinical trials.

Despite these limitations, the broad pharmacological activities demonstrated in preclinical models suggest that *Schisandra chinensis* lignans hold promise as adjunctive therapies for a range of human diseases. To fully realize their therapeutic potential, well-designed clinical trials are needed to evaluate their safety, efficacy and integration into existing treatment regimens. Addressing the current gap in clinical research through rigorously conducted studies will be crucial in determining their translational viability in human health.

## Harnessing the effects of *Schisandra chinensis* lignans

### Combination with other conventional treatments

Emerging evidence highlights the potential of *Schisandra chinensis* lignans to synergize with conventional chemotherapeutic agents, enhancing their efficacy while mitigating drug-induced toxicity. For example, gomisin A has been shown to synergize with paclitaxel to suppress ovarian cancer in vitro and in vivo. This combination reduced ROS levels and inhibited cell cycle progression by downregulating the expression of cyclin-dependent kinase 4 (CDK4) and cyclin B1 [125]. Similarly, schisandrin B has been shown to enhance the efficacy of apatinib in gastric cancer cells. This combination inhibited cell proliferation via G0/G1 phase cell cycle arrest, induced apoptosis by downregulating BAX and caspases-9 and -12, and suppressed cell migration and invasion. These findings highlight the potential of combining schisandrin B with apatinib as an effective therapeutic strategy [126]. Furthermore, schisandrin B has been identified as a promising adjuvant when it is used with 5-fluorouracil (5-FU) in gastric cancer. This combination inhibited cancer cell growth, migration and invasion while reducing STAT3 phosphorylation, inducing autophagy and enhancing 5-FU efficacy both in vitro and in vivo [127]. A recent study by our group also demonstrated that schisandrin B and 5-FU cotreatment resulted in synergistic antitumour effects by improving drug bioavailability, enhancing metabolism and attenuating MDR [128].

Another lignan, schisandrin (schisandrol A), has demonstrated significant potential in overcoming drug resistance in cancer treatment, particularly in the context of P-gp-mediated MDR in cells. Schisandrol enhanced the cytotoxic effects of P-gp substrate cancer drugs, such as vinblastine, by increasing drug retention within cancer cells without altering P-gp expression. This effect was achieved through restoring cell cycle arrest, increasing P-gp-ATPase activity and modifying the interaction between P-gp and the UIC-2 monoclonal antibody. By enhancing substrate binding and reducing drug affinity for P-gp, schisandrol effectively increased the efficacy of P-gp substrate drugs, offering a promising strategy to counteract MDR in cancer treatment [96].

While research on *Schisandra chinensis* lignans has focused predominantly on their anticancer potential, their biological activities suggest a broader scope for therapeutic applications. The ability of these compounds to mitigate oxidative stress, inflammation and metabolic imbalances indicates their potential utility in treating neurodegenerative disorders, cardiovascular diseases, and liver dysfunction. Owing to their potent antioxidant and anti-inflammatory properties, these lignans could complement conventional therapies, enhancing their efficacy and reducing their side effects. Future studies should explore these bioactive compounds in diverse disease contexts to determine their full therapeutic potential. By leveraging their unique mechanisms of action, *Schisandra chinensis* lignans hold considerable promise for expanding their use beyond cancer into a wide range of health conditions.

### Development of novel drug delivery systems

Like many natural products, *Schisandra chinensis* shows increasing promise in the treatment of human diseases because of its bioactive components, particularly lignans. However, their therapeutic efficacy is significantly influenced by pharmacokinetic factors, including absorption, distribution, metabolism and excretion, which directly impact their bioavailability [99]. Many of these compounds are prone to degradation in the GI tract because of factors such as varying pH levels, poor water solubility, rapid metabolism and high clearance rates. To design effective therapies, it is crucial to account for the characteristics of the tissue/tumor microenvironment, tissue/tumor heterogeneity, drug properties, colonic transit time, and pH variations within the GI tract. For example, orally administered *Schisandra chinensis* lignans must endure changes in pH and enzymatic activity as they pass through the GI system before reaching target sites such as colon cancer cells. The ionization state of these bacteria is altered as they transition from the highly acidic environment of the stomach (pH 1–3) to the more neutral pH of the intestine (pH 6–7), which can affect their stability, absorption and overall efficacy [129, 130]. Addressing these challenges is essential for maximizing the therapeutic potential of *Schisandra chinensis* lignans.

To address the critical limitations of *Schisandra chinensis* lignans, advanced drug delivery strategies, particularly nanotechnology-based approaches, have emerged as promising solutions. Nanomedicine enhances drug stability by protecting lignans from enzymatic and chemical degradation while significantly increasing their bioavailability through improved solubility and absorption. Targeted drug delivery systems enable precise accumulation of lignans in specific tissues, such as tumors, inflamed regions, or infected sites, thereby maximizing therapeutic efficacy while minimizing off-target effects. Furthermore, these systems facilitate controlled drug release, ensuring sustained therapeutic action and reducing the dosing frequency. Importantly, nanocarriers reduce toxicity to healthy tissues by limiting systemic exposure and exerting therapeutic effects on diseased areas. These advancements, including nanoparticle-, lipid- or polymer-based carriers, offer a transformative approach to overcoming the bioavailability and stability challenges of *Schisandra chinensis* lignans, paving the way for their broader clinical application.

**Fig. 4:**
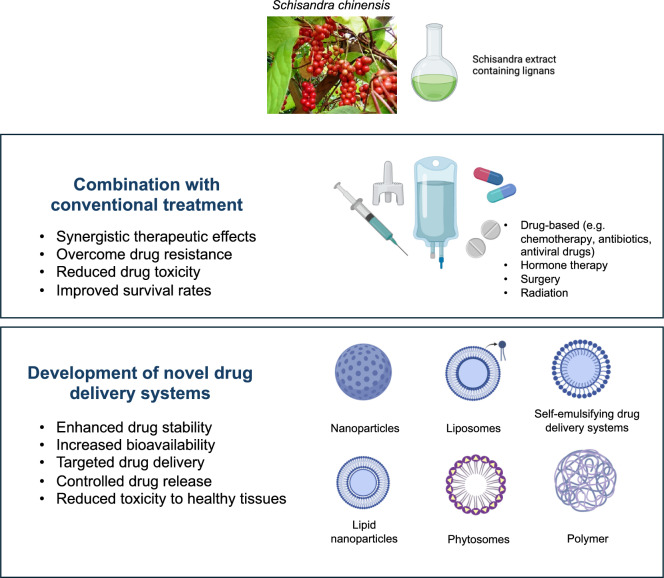
Strategies to increase the bioavailability and effectiveness of *Schisandra chinensis* lignans. The upper panel illustrates the synergistic effects of *Schisandra chinensis* lignans when combined with conventional treatments such as chemotherapy, antibiotics, antiviral drugs, hormone therapy, surgery, and radiation, which contribute to reduced drug toxicity, overcoming drug resistance, and improving survival rates. The lower panel shows the development of novel drug delivery systems, including nanoparticles, liposomes, lipid nanoparticles, phytosomes, polymers and self-emulsifying drug delivery systems, which enhance drug stability, bioavailability, targeted delivery and controlled release while minimizing toxicity to healthy tissues

Some strategies have been proposed/explored to improve the bioavailability of *Schisandra chinensis* lignans (Fig. 4), including the following:

*Nanoparticle-based systems*: These systems employ nanocarriers, including polymeric nanoparticles, solid lipid nanoparticles (SLNs) and inorganic nanoparticles, to encapsulate lignans and enhance their therapeutic efficacy through targeted and controlled release mechanisms. For example, a study prepared *Schisandra* lignan-loaded enteric nanoparticles via a modified spontaneous emulsification solvent diffusion method. The nanoparticles exhibited favorable characteristics, such as appropriate size and drug-loading efficiency. Compared with traditional formulations, in vitro and in vivo evaluations demonstrated increased dissolution rates and improved bioavailability of lignans [131].

*Lipid-based strategies*: Liposomes and phytosomes have emerged as effective strategies to improve the delivery and therapeutic efficacy of *Schisandra* chinensis lignans. Liposomes, vesicles enclosed by phospholipid bilayers, can solubilize water-insoluble compounds such as *Schisandra* lignans into the lipid domain of the liposomal membrane [132]. Studies have shown that liposomal encapsulation of lignans such as schisandrin B enhanced their cytotoxicity against breast cancer cells by effectively inhibiting vasculogenic mimicry (VM) and suppressing tumor invasion and migration through modulation of the expression of key markers such as vascular endothelial growth factor (VEGF), MMP-9, vimentin, and E-cadherin. In vivo pharmacodynamic studies have shown that these liposomal formulations significantly improved antitumor efficacy, with increased tumor cell apoptosis [133]. In another study, deoxyschizandrin-loaded liposomes increased the effect of deoxyschizandrin on adipocytes to reduce their differentiation through effective hepatic targeting, suggesting potential effects on alleviating lipid-associated diseases and nonalcoholic fatty liver disease (NAFLD) [134]. Furthermore, liposomes containing β-cyclodextrin inclusion complexes addressed the poor aqueous solubility and taste issues of *Schisandra* lignans. Pharmacokinetic studies in rats revealed that this formulation increased the plasma concentration and half-life, improving bioavailability and liver-targeting effects [135].

Similarly, phytosomes, which incorporate lignans into a phospholipid matrix, have demonstrated significant improvements in solubility and permeability through biological membranes, enhancing their absorption and systemic delivery [136]. It is expected that phytosomes even offer several advantages over liposomes for delivering *Schisandra chinensis* lignans, particularly owing to their structural integration of lignans into a phospholipid matrix, which enhances bioavailability and stability. Unlike liposomes, which encapsulate hydrophobic lignans in their lipid bilayer, phytosomes form a molecular complex that improves solubility in both aqueous and lipid environments, facilitating better intestinal absorption and systemic delivery [136]. Phytosomes also exhibit greater stability during storage, reducing the risk of oxidation or leakage, and provide sustained release of lignans without requiring additional modifications [137]. These attributes make phytosomes a more efficient and cost-effective drug delivery system for *Schisandra chinensis* lignans.

Self-emulsifying drug delivery systems (SEDDSs) are emerging as promising approaches to increase the solubility and bioavailability of poorly water-soluble compounds. These systems comprise a mixture of oils, surfactants, and cosolvents that spontaneously form fine oil-in-water emulsions upon contact with GI fluids [138]. Studies have shown that lignans incorporated into SEDDSs significantly improve dissolution rates and absorption, bypassing the limitations of poor aqueous solubility. For example, schisandrin or schisandrin B-loaded SEDDSs presented increased GI permeability and increased systemic bioavailability in pharmacokinetic studies. Furthermore, the small droplet size of the emulsions enabled better distribution and absorption while protecting lignans from enzymatic degradation in the GI tract, making SEDDSs an efficient platform for delivering *Schisandra chinensis* lignans in therapeutic applications [139].

*Polymer-based*: Polymer-based drug delivery systems represent a crucial and advantageous approach in the biopharmaceutical industry, offering versatile solutions for the transport of a wide array of therapeutic agents. These systems involve encapsulating lignans within biocompatible and biodegradable polymers, such as poly(lactic-co-glycolic acid) (PLGA), chitosan and alginate, which provide controlled release and targeted delivery [140]. For example, lignans encapsulated in PLGA nanoparticles have shown improved stability and sustained release profiles, ensuring a prolonged therapeutic effect while reducing the dosing frequency [141]. Similarly, chitosan-based nanocarriers have demonstrated enhanced mucoadhesion and GI stability, facilitating better absorption of the encapsulated compounds [142, 143]. These polymer-based systems not only protect lignans from degradation in the GI environment but also allow for surface modifications to enable tissue-specific targeting, such as the liver or brain [144, 145]. Such advancements in polymer-based drug delivery systems may present a versatile and effective approach to harness the full pharmacological potential of *Schisandra chinensis* lignans for treating various diseases.

The development of formulations for *Schisandra* lignans is particularly challenging due to their complex composition, poor water solubility, and rapid metabolism, which affect their stability, bioavailability and therapeutic efficacy. The presence of multiple bioactive lignans with varying physicochemical properties complicates the design of a standardized formulation, making it difficult to achieve consistent pharmacokinetics and predictable therapeutic outcomes. Additionally, lignan mixtures are prone to metabolic transformations that can alter their bioactivity, requiring sophisticated delivery systems to enhance stability and controlled release. Ensuring large-scale production with reproducible quality while meeting regulatory requirements adds another layer of difficulty. Overcoming these challenges requires innovative formulation strategies to enhance solubility, improve absorption and extend circulation time, ultimately facilitating their clinical translation.

## Conclusion and future perspectives

*Schisandra chinensis* has a well-documented history of medicinal use, with a broad spectrum of bioactive compounds—including phenolic acids, lignans, antioxidants, polysaccharides, and triterpenoids—demonstrating promising therapeutic potential. Its wide spectrum of pharmacological activities, including antioxidant, anti-inflammatory, antimicrobial/viral, hepatoprotective, neuroprotective, antidiabetic and anticancer properties highlight its value in disease prevention and treatment. However, despite extensive preclinical research, the clinical translation of *Schisandra chinensis* remains limited by challenges such as poor bioavailability, low water solubility and the instability of its bioactive constituents. These limitations necessitate the development of innovative strategies to enhance its pharmacokinetic properties and therapeutic efficacy.

Nanocarrier-based drug delivery systems have shown considerable promise in preclinical studies for addressing these limitations, but their translation into human clinical trials remains a significant challenge. Many advanced delivery systems are still at an experimental stage, with issues such as scalability, high development costs and complexity hindering broader adoption. Moreover, although *Schisandra chinensis* is generally regarded as safe and has minimal side effects [2], comprehensive studies evaluating its toxicity under pathological conditions and potential interactions with other drugs are lacking. This gap in research underscores the need for more robust in vivo and clinical investigations to ensure safety and efficacy.

The future of *Schisandra chinensis* in disease prevention and treatment hinges on developing more efficient drug delivery systems that can overcome bioavailability and stability issues and gain a deeper understanding of the molecular mechanisms underlying the therapeutic effects of bioactive compounds. Omics technologies, including genomics, proteomics, and metabolomics, will be instrumental in elucidating its mechanisms of action and identifying novel therapeutic targets. Additionally, advances in mass spectrometry and extraction techniques have the potential to refine its pharmacological profile by uncovering previously unidentified bioactive components and optimizing their stability and bioavailability.

By overcoming these barriers, *Schisandra chinensis* holds significant promise for personalized medicine and as an adjunct therapy for cancer and other chronic diseases. Future research should prioritize well-designed, longitudinal clinical trials to assess its long-term safety and efficacy, alongside pharmacokinetic studies to better understand its metabolic pathways and therapeutic impact. With continued scientific advancements, this powerful natural product may yet emerge as a valuable therapeutic agent with widespread clinical applications.

## Data Availability

Not applicable.
